# Implementing patient-reported outcomes in clinical decision-making within knee and hip osteoarthritis: an explorative review

**DOI:** 10.1186/s12891-019-2620-2

**Published:** 2019-05-17

**Authors:** Natasha Lee Sørensen, Lianna Hede Hammeken, Janus Laust Thomsen, Lars Holger Ehlers

**Affiliations:** 1 Danish Center for Healthcare Improvements, Department of Business and Management, Aalborg University, Aalborg, Denmark; 2 Center for General Practice, Department of Clinical Medicine, Aalborg University, Aalborg, Denmark

**Keywords:** Osteoarthritis, Patient-reported outcome measures, Clinical decision-making, Patient participation

## Abstract

**Background:**

In the past few decades, there has been an increasing focus on the importance of patient involvement in the health care system. Patient participation executed through patient-reported outcomes (PROs) and the integration of such into clinical practice has been framed as positive for patients, care providers, and the health care system as a whole. This review aims to elucidate and discuss the current and future use of PROs in clinical practice and to identify the most common types of PRO measures (PROMs) used for patients with hip or knee osteoarthritis in different treatment settings.

**Methods:**

The following databases were searched: PubMed, Embase, CINAHL, Scopus, the Cochrane Library, and EconLit. For inclusion in the study, studies had to cover either knee or hip osteoarthritis and report on PROs. The type of PROM, treatment setting, and study design of each included study were extracted from their respective abstracts. Additionally, the full text of studies concerning PROs as an integrated part of clinical practice was examined and information on the year of publication, study design, topic, and use of PROMs was extracted.

**Results:**

It was found that only two pilot studies reported on the use of PROs as an integrated part of patient treatment within hip or knee osteoarthritis. In 349 studies, a total of 38 different PROMs relevant for patients with either hip or knee osteoarthritis were identified. The EQ-5D, WOMAC, and VAS questionnaires were the most commonly reported generic, disease-specific, and domain-specific PROMs, respectively. However, a large variation in the use of different PROMs both within and between surgical and nonsurgical settings was found.

**Conclusion:**

Limited evidence on the use of PROs as an integrated part of clinical practice for patients with hip and knee osteoarthritis was found. Further research is necessary to clarify the effects on patient outcomes of using PROs in clinical practice. In addition, there is limited agreement on a joint standard for the use of PROMs both within and across the sectorial boarders. Further exploration of PROMs to generate future standardisation is suggested.

**Electronic supplementary material:**

The online version of this article (10.1186/s12891-019-2620-2) contains supplementary material, which is available to authorized users.

## Background

In the past few decades, there has been an increasing focus on the importance of patient involvement in the health care system. Patient participation executed through patient-reported outcomes (PROs) has been framed as being positive for the patients, care providers, as well as the health care system as a whole. [1, 2] PROs can be defined as measures of a patient’s state of health that come directly from the patient, without interpretation by others [3, 4]. Until recently, PROs have primarily been used for research purposes in clinical trials. However, interest is growing regarding integrating collection of PROs directly into clinical practice [5–8]. In this context, PROs hold a potential for facilitating shared decision-making by promoting patient involvement in their own treatment [5–8]. In addition, the integration of PROs into clinical practice can help to inform on indications for different treatment options and can be used to monitor the effectiveness of a treatment [7]. PROs are measured systematically using tools or instruments sometimes referred to as PRO measures (PROMs) [4], which can be of either a generic, disease-specific, or domain-specific character [4, 9]. Generic questionnaires, often measuring the quality of life (QoL) can be aggregated across different types of diseases. Disease-specific questionnaires focus mainly on a particular disease or condition, whereas domain-specific questionnaires aim at measuring specific domains of a disease, e.g. pain [4, 9].

A number of reviews have been conducted to date to evaluate the effects of using PROs as an integrated part of clinical treatment [7, 10–12]. These reviews generally have found insufficient evidence to support the implementation of PROs in clinical practice. However, to the authors’ knowledge, no systematic evaluations investigating the use of PROs in clinical practice within hip or knee osteoarthritis (OA) have been performed previously [10, 11]. First-line treatment of OA consists of nonsurgical treatment [13]. When OA is advanced and no other treatment is efficient, referral for surgical treatment is considered [13]. For patients with OA, information on PROs like pain and daily function is important for diagnostic and treatment considerations [14–16]. Furthermore, not fulfilling patient expectations has been found to be negatively correlated with satisfaction after total knee arthroplasty [17, 18]. Therefore, using PROs to improve shared decision-making and the matching of expectations might improve postsurgery patient satisfaction. As a result, using PRO as an integrated part of clinical practice can be highly relevant within hip and knee OA. Accordingly, in this review, it was aimed to investigate and discuss the current and future use of PROs in clinical practice and to identify the most common types of PROMs used for patients with hip or knee OA in different treatment settings.

## Methods

The reporting of this explorative review follows the Preferred Reporting Items for Systematic Reviews and Meta-Analyses (PRISMA) guidelines, where applicable [19].

### Data sources

All relevant studies were identified in a systematic literature search. The following databases were searched: PubMed, Embase, CINAHL, Scopus, the Cochrane Library and EconLit. The search was conducted on April 5, 2018. For inclusion in the present review, the studies had to (1) concern either knee or hip OA and (2) report on PROs. The search strategy consisted of several different keywords for OA and PRO, which were combined by use of the Boolean operators ‘OR’ and ‘AND’. Regarding PRO, the search was limited to search in title and abstracts. No restrictions regarding the year of publication, language, or publication status were applied and no grey literature search was conducted. The full electronic search strategy can be found in Additional file 1.

### Study selection

The process for study selection consisted of the removal of duplicates and the exclusion of ineligible studies. Studies were excluded if they were systematic reviews, did not examine OA, and/or did not mention any PROMs. Two authors (NLS and LHH) conducted a preliminary screening of 100 studies (title and abstract) to identify themes of study design and to validate their approach to screening based on the eligibility criteria. The two authors achieved a 95% agreement for the inclusion of studies in the preliminary screening. After the preliminary screening, the full number of identified studies was divided in half and each author screened half of the studies. Only the abstracts of the studies were examined.

### Data extraction

The following information was extracted from the abstracts: (1) type(s) of PROMs reported in the study and (2) the setting of each study, identified as either surgical, nonsurgical, both surgical and nonsurgical, or no specified treatment reported. In addition, each study was assigned a theme of study design categorized as either (1) a validation study (e.g., PROMs validated in a specific setting); (2) a study on clinical effect (e.g., one surgical approach compared with another by means of PROs); (3) a prediction study (e.g., a test on different parameters’ effects on PROs); (4) a study on PROs as an integrated part of clinical practice; or (4) a study design different from the other categories (e.g., studies on minimally important differences). A random sample of 20% of the studies that were considered eligible for inclusion was examined in full text by two pairs of two authors. This was done to evaluate whether any of the studies that were assigned to another theme, was actually reporting on PROs as an integrated part of clinical practice. In addition, the full text of all studies concerning PROs as an integrated part of clinical practice was examined. Information on the year of publication, study design, topic, and use of PROMs was extracted. The study was explorative and there was no formal assessment of the quality of the included studies. Data were extracted to a template made in Microsoft Excel (2016) (Microsoft Corp., Redmond, WA, USA).

## Results

**Fig. 1:**
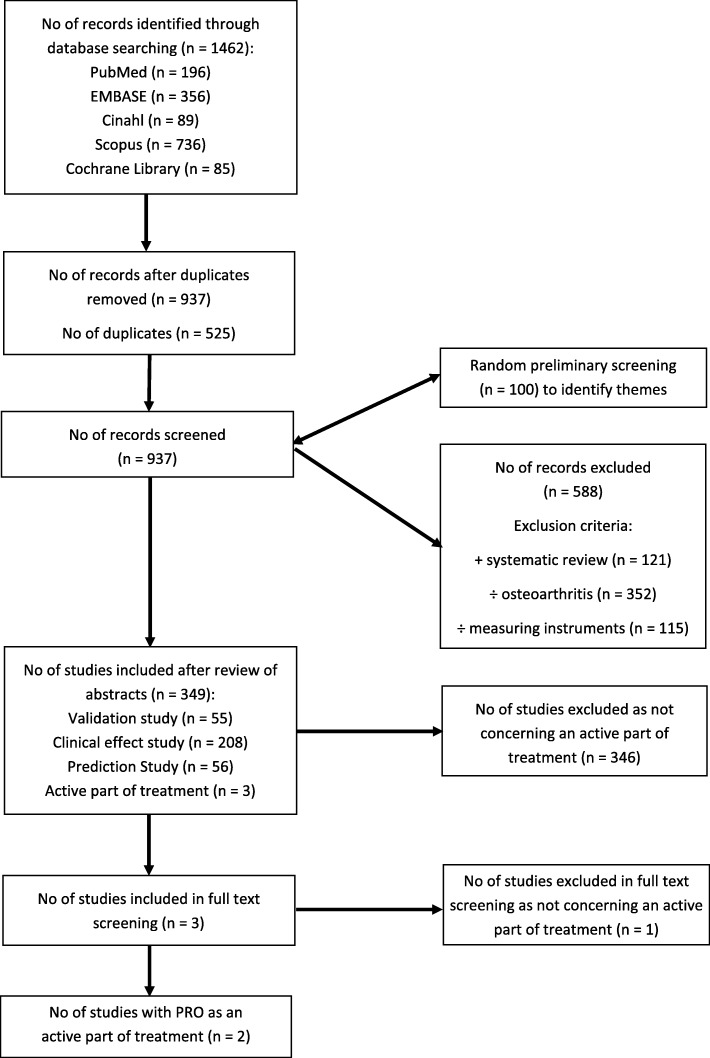
Flow-diagram of the study selection

The performed systematic literature search identified 1462 studies (Fig. 1). Five hundred twenty-five duplicates were removed, which resulted in the further screening of 937 studies. From this, 588 studies were excluded; thus, in total, 349 studies were included in the present review. Of these studies, three studies were identified for full-text screening. Full-text screening resulted in the exclusion of one study, as it was classified as a prediction study, and the final inclusion of two studies concerning the use of PROs as an integrated part of clinical practice.

### Results of data synthesis

A total of 38 different PROMs relevant for patients with hip or knee OA were reported within the 349 identified studies (Additional file 2); of these, six PROMs were identified as generic, while 24 and eight PROMs, respectively, were identified as disease-specific or domain-specific. Additional file 3 describes the identified PROMs and the categorization. Table 1 presents the number of times each type of PROM was reported. The 38 identified PROMs were reported a total number of 684 times in the 349 included studies, corresponding to a rate of approximately two PROMs reported in each study. Generic PROMs were reported in 25.9% of the cases, while domain-specific PROMs were reported in 6.6% of the cases. Disease-specific PROMs were the most commonly reported PROMs in the published literature within hip and knee OA, being reported in 67.5% of the cases.

**Table 1:** An overview of the different types of PROMs reported in the 349 identified studies

Type of PROM	Number of different PROMs	Number of times reported (%)
Generic	6	177 (25.9)
Disease-specific	24	462 (67.5)
Domain-specific	8	45 (6.6)
Total	38	684 (100)

Table 2 illustrates the number of times each PROM was reported across different settings. Considering generic PROMs, the most frequently reported PROMs were the European Quality of Life-5 Dimensions (EQ-5D), Short Form-12 (SF-12), and Short Form-36 (SF-36) questionnaires reported in 35.6, 29.9, and 26.0% of the cases, respectively. Of note, the EQ-5D (43.7%) and SF-12 (32.8%) were the most commonly reported generic PROMs within a surgical setting. In contrast, considering a nonsurgical setting, the four most applied generic PROMs (i.e., EQ-5D, SF-12, SF-36, and Patient-Reported Outcomes Measurement Information System (PROMIS)) were used approximately an equal number of times (20.6–26.5%). For the disease-specific PROMs, the most commonly reported one was the Western Ontario and McMaster Universities Osteoarthritis Index (WOMAC), used in more than one-quarter (26.4%) of the cases. WOMAC was the most frequently reported disease-specific PROM within both the surgical and nonsurgical settings. However, the variation in the use of PROMs was greater within the surgical setting, ranging between 11.8 and 22.3% for the five most commonly applied PROMs (i.e., WOMAC, Oxford Knee Score (OKS), Knee Injury and Osteoarthritis Outcome Score (KOOS), Knee Society Score (KSS), and Oxford Hip Score (OHS)), as compared with in the nonsurgical setting, in which WOMAC and KOOS were used in more than two-thirds (67.3%) of the cases. For the domain-specific PROMs, the Visual Analogue Scale (VAS) was reported in nearly three-quarters (73.3%) of the cases, and no significant variation was found between the PROMs used in the surgical versus nonsurgical settings.

**Table 2:** The use of PROMs in different settings within knee and hip OA

Type of PROM^a^	Surgical: n (%)	Non-surgical: n (%)	Both surgical and non-surgical: n (%)	Unspecified treatment: n (%)	Total: n (%)
Generic					
EQ-5D	52 (43.7)	7 (20.6)	3 (33.3)	1 (6.7)	63 (35.6)
SF-12	39 (32.8)	9 (26.5)	1 (11.1)	4 (26.7)	53 (29.9)
SF-36	27 (22.7)	7 (20.6)	5 (55.6)	7 (46.7)	46 (26.0)
PROMIS	0 (0)	8 (23.5)	0 (0)	3 (20.0)	11 (6.2)
Other^b^	1 (0.8)	3 (8.8)	0 (0)	0 (0)	4 (2.3)
Subtotal	119 (100)	34 (100)	9 (100)	15 (100)	177 (100)
Disease-specific					
WOMAC	70 (22.3)	23 (39.7)	9 (34.6)	20 (31.3)	122 (26.4)
KOOS	37 (11.8)	16 (27.6)	5 (19.2)	14 (21.9)	72 (15.6)
OKS	57 (18.2)	3 (5.2)	2 (7.7)	4 (6.3)	66 (14.3)
KSS	45 (14.3)	1 (1.7)	0 (0)	2 (3.1)	48 (10.4)
OHS	37 (11.8)	0 (0)	2 (7.7)	1 (1.6)	40 (8.7)
HOOS	24 (7.6)	3 (5.2)	3 (11.5)	5 (7.8)	35 (7.6)
HHS	19 (6.1)	0 (0)	4 (15.4)	3 (4.7)	26 (5.6)
FJS	17 (5.4)	0 (0)	0 (0)	0 (0)	17 (3.7)
Other^c^	8 (2.5)	12 (20.7)	1 (3.8)	15 (23.4)	36 (7.8)
Subtotal	314 (100)	58 (100)	26 (100)	64 (100)	462 (100)
Domain-specific					
VAS	16 (88.9)	9 (69.2)	2 (100)	6 (50)	33 (73.3)
Other^d^	2 (11.1)	4 (30.8)	0 (0)	6 (50)	12 (26.7)
Subtotal	18 (100)	13 (100)	2 (100)	12 (100)	45 (100)
Total	451	105	37	91	684

^a^The full names of the PROMs are available in Additional file 2.

^b^SANE, PGI-S/C

^c^LEFS, TLKS, IKDC, ASES, KOS, PGAP (O2MS), ICOAP, LAI, HAAS, JHEQ, AKPS (KUJALA), OAQOL, OAHQOL (AMIQUAL), HOS, IHOT-33, ASAP

^d^BPI, NRS, PD-Q, PIQ, PASE, CPGS, LATE-LIFE DFI

In Table 3, the distribution of studies across each stated theme is presented in relation to treatment settings. The classification of study designs revealed that clinical effect was the most commonly applied theme among the studies (59.6%), followed by prediction studies (16.3%) and validation studies (15.8%). Only two (0.6%) of the studies reported on PROs as an integrated part of clinical practice. Furthermore, 7.7% of the studies reported on a different theme that did not fit into the established categories. Table 3 also reveals that the majority of PROMs noted were reported in studies covering surgical treatment (65.9%) versus nonsurgical treatment (15.2%), a combination of surgical and nonsurgical treatment (5.7%), and unspecified treatment (13.2%).

**Table 3:** The types of study themes and treatment settings in which PROMs were used

Study type	Surgical: n (%)	Non-surgical: n (%)	Both surgical and non-surgical: n (%)	Unspecified treatment: n (%)	Total: n (%)
Validation	23 (6.6)	8 (2.3)	1 (0.3)	23 (6.6)	55 (15.8)
Clinical effect	148 (42.4)	35 (10.0)	17 (4.9)	8 (2.3)	208 (59.6)
Prediction model	46 (13.2)	5 (1.4)	2 (0.6)	4 (1.1)	57 (16.3)
PRO as an active part of the treatment	1 (0.3)	0 (0)	0 (0)	1 (0.3)	2 (0.6)
Other studies	12 (3.4)	5 (1.4)	0 (0)	10 (2.9)	27 (7.7)
Total	230 (65.9)	53 (15.2)	20 (5.7)	46 (13.2)	349 (100)

The studies reporting on PROs as an integrated part of clinical practice within hip or knee OA are presented in Table 4. The study by Gakhal et al. [20] is a conference abstract concerning the development and implementation of a system for PRO feedback to encourage the use of data to inform clinical decisions by patients and their providers. Furthermore, the study by Slover et al. [21] is a feasibility study with the aim of evaluating the integration of an electronic PRO data capture system into routine orthopaedic practice.

**Table 4:** The identified studies in which PROs as an integrated part of clinical practice were examined

Study	Title	Year	Study design	Topic	PROM^a^
Gakhal N et al. [20]	Audit and feedback of patient reported outcomes in knee osteoarthritis to improve management in primary care: A pilot project	2016	Pilot study	Implementation of a system of feedback of PRO to encourage the use of data to inform clinical decisions by patients and their providers.	ICOAP, WOMAC
Slover JD, et al. [21]	Feasibility of integrating standardized patient-reported outcomes in orthopedic care	2015	Feasibility study	Demonstration of the feasibility of routinely collecting PRO as a part of standard orthopaedic care.	EQ-5D, KOOS

^a^The full names of the PROMs are available in Additional file 2

## Discussion

Integrating PROs into clinical practice has previously been proposed as a potential way to improve shared decision-making and patients’ involvement in their own treatment. However, there is currently a very limited amount of evidence regarding the active use of PROs within hip or knee OA. In this explorative review, only two pilot/feasibility studies reported on the integration of PROs in clinical practice to improve patient outcomes. PROs were primarily reported in studies regarding clinical effect, followed by studies on prediction models and validation studies. In 349 studies, a total of 38 different PROMs relevant for patients with either hip or knee OA were identified. The EQ-5D, WOMAC, and VAS questionnaires were the most commonly reported generic, disease-specific, and domain-specific PROMs, respectively. However, a large variation in the use of PROMs both within and between surgical and nonsurgical settings was found.

In this investigation, the literature search performed was broad, with the aim of including all available studies found concerning PRO within hip and knee OA. Due to the explorative nature of this review, a formal assessment of the quality of the included studies was not conducted. However, some limitations of the review process exist. Even though several databases of scientific literature were searched for this review, no search of the grey literature was conducted. It is acknowledged that PROs are applied within quality improvement efforts and in the maintenance of health care services [22] and some studies could therefore have been missed. However, the aim of the current review was to identify scientific research studies on the use of PROs, which is why the omission of grey literature is considered of limited importance. Also, for the majority of the studies, only abstracts and not full-text studies were reviewed, leading to a possible bias regarding the inclusion and classification of studies, as relevant information could have been overlooked. At last, the screening of most studies was performed by separating the total number of studies in two halves, with each half screened by one reviewer (two reviewers total). As a result, there is a risk that the two reviewers made or would have made different decisions from one another regarding the eligibility and classification of the studies they reviewed. However, to limit the risk of bias, both reviewers participated in a preliminary screening of the first 100 titles and abstracts and compared and discussed the decisions of eligibility and, from this, a high level of agreement (95%) was achieved. Furthermore, a random sample of 20% of the studies considered eligible for inclusion during the screening of title and abstract (70 studies) were assessed in full text by the four authors in two pairs of two. The authors agreed that none of the 70 studies in the secondary screening concerned PROs as an integrated part of clinical practice, thereby increasing the confidence in the categorisation of the studies.

In this explorative review, only two studies reporting on the active use of PROs as an integrated part of clinical practice within hip and knee OA were identified. Gakhal et al. [20] reported on the effects of continuous feedback provided to doctors about their patients’ pain and functional level. Separately, Slover et al. [21] described the integration of a uniform, web-based software system at two different treatment sites to create a multisite registry of secure PRO data that are usable in real time by both patients and physicians. Over the last 10 years, a number of studies [10, 11], have examined the use and application of PROs as an integrated part of clinical practice within different clinical areas and with varying conclusions. Importantly, the integration of PROs into clinical practice can be complex and resource-intensive [7, 10, 23]. As a result, Porter et al. developed a framework in which six key issues were highlighted. When considering to integrate PRO into clinical practice it is suggested to clarify the clinical activity that PROs are aimed at improving; to consider the characteristics of the PROM, the feedback system, the setting, and the target groups; and to support the implementation of PRO [7] Considering all of these requirements, it is not surprising that the integration and research into the effectiveness of PROs as a part of clinical practice can be challenging and that the results of interventions can and have differed. Based on the existing inconclusive evidence and the limited number of identified studies within hip and knee OA, more research on the application of PROs in clinical practice is advised to clarify whether integrating PROs into clinical practice works, for whom it works, and under which specific circumstances it works.

Previous systematic reviews, [24–26], have reported on the trends and use of different PROMs within total joint arthroplasty, but, to the authors’ knowledge, no other studies have examined the application of PROMs on patients with hip or knee OA across different settings or the use of PROs integrated in clinical practice. In accordance with the findings in the present study, previous systematic reviews have reported on a larger number of available PROMs within joint arthroplasty. One such systematic review by Ramkumar et al. [24] reported on the psychometric properties and variation among available PROMs within total knee arthroplasty. A total of 47 different PROMs were identified; however, it was concluded that no single ideal PROM was available. This finding was supported by Gagnier et al. [25], who identified 32 PROMs but concluded that many PROMs have limited evidence regarding their psychometric properties. Furthermore, in the present review, a large variation in the use of PROMs, both between and within settings, was identified. Siljander et al. [26] had the objective of analysing trends in the reporting of PROMs within total joint arthroplasty in four major orthopaedic journals. The study identified 42 different PROMs and found that the choice of PROM varied depending on the specific journal, time, treatment procedure, and geographic region. These findings support a hypothesis that suggests that the choice of PROM is currently arbitrary and dependent on traditions rather than based on standards and evidence of the best measuring properties. As a consequence, further exploration of PROMs to generate future standardisation is incited.

In addition to the suggested generation of standardisation within a surgical setting, the authors of the present review suggest that the possibility of standardisation across both surgical and nonsurgical settings should be investigated. The apparent advantages of standardisation across sectorial boarders would be to improve the communication ongoing between different practitioners and to optimise patient flow across sectors. In addition, a cross-sectorial PROM could be beneficial in the use of PROs as an integrated part of the treatment of hip or knee OA. However, it is acknowledged that various practitioners might have different needs and demands for information to provide the best possible treatment to the patient and that the importance of involving practitioners in the development of a cross-sectorial tool must be emphasised.

## Conclusion

The aim of this explorative review was to investigate and discuss the current and future use of PROs in clinical practice and to identify the most common types of PROMs used for patients with hip or knee OA in different treatment settings. It is now known that limited evidence on the use of PROs as an integrated part of clinical practice for patients with hip or knee OA exist. It is suggested that further primary research will be necessary to clarify the effectiveness of integrating PROs into clinical practice within hip or knee OA before a systematic review with a risk of bias appraisal of the included studies will be relevant. Furthermore, it was found that the EQ-5D, WOMAC and VAS questionnaires were the most commonly reported generic, disease-specific, and domain-specific PROMs, respectively. However, it was also found that many different PROMs are utilised in studies concerning knee or hip OA. This suggests that there is limited agreement on a joint standard for the use of PROMs within hip or knee OA both within and across sectorial borders. The choice of PROM might be arbitrary and dependent on traditions rather than on standards and evidence of the best measuring properties. It is recommended that further research should investigate the possibilities for future standardisation of PROMs within hip or knee OA.

## Additional files

Additional file 1: is a .doc file which contains information on the full electronic search strategy. (DOCX 17 kb)

Additional file 2: is a .doc file which contains information on the 349 included studies. (DOCX 55 kb)

Additional file 3: is a .doc file which describes the included patient-reported outcome measures, abbreviations and classifications of the PROMs as generic, disease-specific or domain-specific questionnaires. (DOCX 35 kb)

## Abbreviations

EQ-5D: European Quality of Life-5 Dimensions
KOOS: Knee Injury and Osteoarthritis Outcome Score
KSS: Knee Society Score
OA: osteoarthritis
OHS: Oxford Hip Score
OKS: Oxford Knee Score
PRO: Patient-reported outcome
PROM: patient-reported outcome measure
PROMIS: Patient-Reported Outcomes Measurement Information System
SF-12: Short Form-12
SF-36: Short Form-36
VAS: Visual Analogue Scale
WOMAC: Western Ontario and McMaster Universities Osteoarthritis Index
