# Supplementary material for: Implementing patient-reported outcomes in clinical decision-making within knee and hip osteoarthritis: an explorative review
Source: BMC Musculoskelet Disord. 2019 May 17;20:230. doi: 10.1186/s12891-019-2620-2 (PMC6525425; doi:10.1186/s12891-019-2620-2)
Supplement: Supplementary file 1 — is a .doc file which contains information on the full electronic search strategy. (DOCX 17 kb) [file 12891_2019_2620_MOESM1_ESM.docx]

# Additional file 1

##

## PubMed

("Osteoarthritis, Knee"[All Fields] OR "knee osteoarthritis"[All Fields] OR "knee arthrosis"[All Fields] OR "knee joint arthrosis"[All Fields] OR "Osteoarthritis, Hip"[All Fields] OR "hip osteoarthritis"[All Fields] OR "hip osteoarthrosis"[All Fields] OR "coxarthrosis"[All Fields] OR "hip arthrosis"[All Fields] OR "hip joint arthrosis"[All Fields]) AND ("patient reported outcome*"[Title/Abstract] OR "patient reported outcome measure*"[Title/Abstract] OR "patient-reported outcome*"[Title/Abstract] OR "patient-reported outcome measure*"[Title/Abstract])

## Embase

('knee osteoarthritis' OR 'knee osteoarthrosis' OR 'knee arthrosis' OR 'knee joint arthrosis' OR 'hip osteoarthritis' OR 'hip osteoarthrosis' OR 'coxarthrosis' OR 'hip arthrosis' OR 'hip joint arthrosis') AND ('patient-reported outcome*':ab,ti OR 'patient reported outcome*':ab,ti OR 'patient-reported outcome measure*':ab,ti OR 'patient reported outcome measure*':ab,ti)

## Cinahl

Not possible to combine abstract and title. Search only on abstract

(TX “osteoarthritis, knee” OR TX “knee osteoarthritis” OR TX “knee osteoarthrosis” OR TX “knee arthrosis” OR TX “knee joint arthrosis” OR TX “osteoarthritis, hip” OR TX “hip osteoarthritis” OR TX “hip osteoarthrosis” OR TX “coxarthrosis” OR TX “hip arthrosis” OR TX “hip joint arthrosis”) AND (AB "patient reported outcome*" OR AB "patient-reported outcome*" OR AB "patient reported outcome measure*" OR AB "patient-reported outcome measure*")

## Scopus

( ALL ( "knee osteoarthritis" OR "knee osteoarthrosis" OR "knee arthrosis" OR "knee joint arthrosis" OR "hip osteoarthritis" OR "hip osteoarthrosis" OR "coxarthrosis" OR "hip arthrosis" OR "hip joint arthrosis" ) ) AND ( TITLE-ABS-KEY ( "patient reported outcome*" OR "patient reported outcome measure*" OR "patient-reported outcome*" OR "patient-reported outcome measure*" ) )

## Cochrane Library

("Osteoarthritis, Knee" or "knee osteoarthritis" or "knee arthrosis" or "knee joint arthrosis" or "Osteoarthritis, Hip" or "hip osteoarthritis" or "hip osteoarthrosis" or "coxarthrosis" or "hip arthrosis" or "hip joint arthrosis") AND ("patient reported outcome*" or "patient reported outcome measure*" or "patient-reported outcome*" or "patient-reported outcome measure*"):ti,ab,kw

## EconLit

("knee osteoarthritis" OR "knee osteoarthrosis" OR "knee arthrosis" OR "knee joint arthrosis" OR "hip osteoarthritis" OR "hip osteoarthrosis" OR "coxarthrosis" OR "hip arthrosis" OR "hip joint arthrosis") AND ("patient-reported outcome*" OR "patient reported outcome*" OR "patient-reported outcome measure*" OR "patient reported outcome measure*")
