# Supplementary material for: Implementing patient-reported outcomes in clinical decision-making within knee and hip osteoarthritis: an explorative review
Source: BMC Musculoskelet Disord. 2019 May 17;20:230. doi: 10.1186/s12891-019-2620-2 (PMC6525425; doi:10.1186/s12891-019-2620-2)
Supplement: Supplementary file 2 — is a .doc file which contains information on the 349 included studies. (DOCX 55 kb) [file 12891_2019_2620_MOESM2_ESM.docx]

Additional file 2

| **Validation study** |
| --- |
| Arbab, D., van Ochten, J.H.M., Schnurr, C., Bouillon, B. & König, D. 2017, "Assessment of reliability, validity, responsiveness and minimally important change of the German Hip dysfunction and osteoarthritis outcome score (HOOS) in patients with osteoarthritis of the hip", *Rheumatology international,* vol. 37, no. 12, pp. 2005-2011. |
| Baumann, F., Ernstberger, T., Loibl, M., Zeman, F., Nerlich, M. & Tibesku, C. 2016, "Validation of the German Forgotten Joint Score (G-FJS) according to the COSMIN checklist: does a reduction in joint awareness indicate clinical improvement after arthroplasty of the knee?", *Archives of orthopaedic and trauma surgery,* vol. 136, no. 2, pp. 257-264. |
| Bellamy, N., Hendrikz, J. & Wilson, C. 2011, "Observations on module 4 (Patient Global Assessment) of the osteoarthritis modular measurement system (O2MSTM)", *Internal Medicine Journal,* vol. 41, pp. 23. |
| Broderick, J.E., Schneider, S., Junghaenel, D.U., Schwartz, J.E. & Stone, A.A. 2013, "Validity and reliability of patient-reported outcomes measurement information system instruments in osteoarthritis", *Arthritis Care and Research,* vol. 65, no. 10, pp. 1625-1633. |
| Chang, F.-., Jette, A.M., Slavin, M.D., Baker, K., Ni, P. & Keysor, J.J. 2018, "Detecting functional change in response to exercise in knee osteoarthritis: A comparison of two computerized adaptive tests", *BMC Musculoskeletal Disorders,* vol. 19, no. 1. |
| Conrozier, T., Monet, M., Lohse, A. & Raman, R. 2017, "Getting Better or Getting Well? The Patient Acceptable Symptom State (PASS) Better Predicts Patient's Satisfaction than the Decrease of Pain, in Knee Osteoarthritis Subjects Treated with Viscosupplementation", *Cartilage,* , pp. 1947603517723072. |
| Cotofana, S., Wirth, W., Rossi, C.P., Eckstein, F. & Günther, O.H. 2015, "Contralateral knee effect on self-reported knee-specific function and global functional assessment: Data from the osteoarthritis initiative", *Arthritis Care and Research,* vol. 67, no. 3, pp. 374-381. |
| Couraud, G., Escalas, C., Etcheto, A., Rannou, F. & Poiraudeau, S. 2015, "French adaptation and validation of the Osteoarthritis Quality of Life scale", *Annals of Physical and Rehabilitation Medicine,* vol. 58, no. 6, pp. 336-342. |
| Dawson, J., Beard, D.J., McKibbin, H., Harris, K., Jenkinson, C. & Price, A.J. 2014, "Development of a patient-reported outcome measure of activity and participation (the OKSAPQ) to supplement the Oxford knee score", *Bone and Joint Journal,* vol. 96 B, no. 3, pp. 332-338. |
| Debette, C., Parratte, S., Maucort-Boulch, D., Blanc, G., Pauly, V., Lustig, S., Servien, E., Neyret, P. & Argenson, J.N. 2014, "French adaptation of the new Knee Society Scoring System for total knee arthroplasty", *Orthopaedics & traumatology, surgery & research : OTSR,* vol. 100, no. 5, pp. 531-534. |
| Diesinger, Y. & Jenny, J.-. 2014, "Validation of the French version of two on high-activity knee questionnaires", *Orthopaedics and Traumatology: Surgery and Research,* vol. 100, no. 5, pp. 535-538. |
| Driban, J.B., Morgan, N., Price, L.L., Cook, K.F. & Wang, C. 2015, "Patient-Reported Outcomes Measurement Information System (PROMIS) instruments among individuals with symptomatic knee osteoarthritis: A cross-sectional study of floor/ceiling effects and construct validity", *BMC Musculoskeletal Disorders,* vol. 16, no. 1. |
| Ebrahimzadeh, M.H., Makhmalbaf, H., Birjandinejad, A. & Soltani-Moghaddas, S.H. 2014, "Cross-cultural adaptation and validation of the persian version of the oxford knee score in patients with knee osteoarthritis", *Iranian journal of medical sciences,* vol. 39, no. 6, pp. 529-535. |
| Gandek, B., Ware, J.E. & Ware, J.E.,Jr 2017, "Validity and Responsiveness of the Knee Injury and Osteoarthritis Outcome Score: A Comparative Study Among Total Knee Replacement Patients", *Arthritis Care & Research,* vol. 69, no. 6, pp. 817-825. |
| Ghomrawi, H.M.K., Mandl, L.A., Rutledge, J., Alexiades, M.M. & Mazumdar, M. 2011, "Is there a role for expectation maximization imputation in addressing missing data in research using WOMAC questionnaire? Comparison to the Standard mean approach and a tutorial", *BMC Musculoskeletal Disorders,* vol. 12, no. 1. |
| Giesinger, J.M., Hamilton, D.F., Jost, B., Behrend, H. & Giesinger, K. 2015, "WOMAC, EQ-5D and Knee Society Score Thresholds for Treatment Success After Total Knee Arthroplasty", *Journal of Arthroplasty,* vol. 30, no. 12, pp. 2154-2158. |
| Giesinger, J.M., Kuster, M.S., Behrend, H. & Giesinger, K. 2013, "Association of psychological status and patient-reported physical outcome measures in joint arthroplasty: A lack of divergent validity", *Health and Quality of Life Outcomes,* vol. 11, no. 1. |
| Giesinger, J.M., Kuster, M.S., Holzner, B. & Giesinger, K. 2013, "Development Of A Computer-Adaptive Version Of The Forgotten Joint Score", *Journal of Arthroplasty,* vol. 28, no. 3, pp. 418-422. |
| Giesinger, K., Hamilton, D.F., Jost, B., Holzner, B. & Giesinger, J.M. 2014, "Comparative responsiveness of outcome measures for total knee arthroplasty", *Osteoarthritis and Cartilage,* vol. 22, no. 2, pp. 184-189. |
| Gudbergsen, H., Bartels, E.M., Krusager, P., Wæhrens, E.E., Christensen, R., Danneskiold-Samsøe, B. & Bliddal, H. 2011, "Test-retest of computerized health status questionnaires frequently used in the monitoring of knee osteoarthritis: A randomized crossover trial", *BMC Musculoskeletal Disorders,* vol. 12. |
| Gudbergsen, H.R. 2010, "Validation of digitalized Health Status Questionnaires frequently used in the monitoring of osteoarthritis: A cross sectional study", *Osteoarthritis and Cartilage,* vol. 18, pp. S147. |
| Haragus, H., Prejbeanu, R., Poenaru, D.V., Deleanu, B., Timar, B. & Vermesan, D. 2018, "Cross-cultural adaptation and validation of a patient-reported hip outcome score", *International orthopaedics,* , pp. 1-6. |
| Harris, K., Dawson, J., Doll, H., Field, R.E., Murray, D.W., Fitzpatrick, R., Jenkinson, C., Price, A.J. & Beard, D.J. 2013, "Can pain and function be distinguished in the Oxford Knee Score in a meaningful way? An exploratory and confirmatory factor analysis", *Quality of Life Research,* vol. 22, no. 9, pp. 2561-2568. |
| Hung, M., Hon, S.D., Cheng, C., Franklin, J.D., Aoki, S.K., Anderson, M.B., Kapron, A.L., Peters, C.L. & Pelt, C.E. 2014, "Psychometric evaluation of the lower extremity computerized adaptive test, the modified harris hip score, and the hip outcome score", *Orthopaedic Journal of Sports Medicine,* vol. 2, no. 12. |
| Ingelsrud, L.H., Terwee, C.B., Gonc¸alves, R.S. & Roos, E.M. 2014, "Minimal important change for the knee injury and osteoarthritis outcome Score (KOOS) in patients with knee osteoarthritis", *Osteoarthritis and Cartilage,* vol. 22, pp. S179-S180. |
| Jenny, J.-. & Diesinger, Y. 2012, "The Oxford Knee Score: Compared performance before and after knee replacement", *Orthopaedics and Traumatology: Surgery and Research,* vol. 98, no. 4, pp. 409-412. |
| Kersten, P., White, P.J. & Tennant, A. 2010, "The Visual Analogue WOMAC 3.0 scale - Internal validity and responsiveness of the VAS version", *BMC Musculoskeletal Disorders,* vol. 11. |
| Klokker, L., Bandak, E., Bartholdy, C., Bliddal, H. & Henriksen, M. 2014, "Pain in activity evaluation (PACE) in knee osteoarthritis-responsiveness and concurrent validity of tentative measures in a randomized controlled exercise study", *Osteoarthritis and Cartilage,* vol. 22, pp. S187. |
| Kumar, M., Battepathi, P. & Bangalore, P. 2015, "Expectation fulfilment and satisfaction in total knee arthroplasty patients using the 'PROFEX' questionnaire", *Orthopaedics and Traumatology: Surgery and Research,* vol. 101, no. 3, pp. 325-330. |
| Lee, A.C., Price, L.L., Driban, J.B., Harvey, W.F., McAlindon, T.E., Rodday, A.M., Knopp, H.E. & Wang, C. 2016, "Longitudinal construct validity for four patient-reported outcomes measurement information system (PROMIS) short forms: Physical function, pain interference, depression, and anxiety among adults with knee osteoarthritis", *Arthritis and Rheumatology,* vol. 68, pp. 3059-3060. |
| Lin, F.-., Samp, J., Munoz, A., Wong, P.S. & Pickard, A.S. 2014, "Evaluating change using patient-reported outcome measures in knee replacement: The complementary nature of the EQ-5D index and VAS scores", *European Journal of Health Economics,* vol. 15, no. 5, pp. 489-496. |
| Lin, K., Bao, L., Wang, J., Fujita, K., Makimoto, K. & Liao, X. 2017, "Validation of the Chinese (Mandarin) Version of the Oxford Knee Score in Patients with Knee Osteoarthritis", *Clinical orthopaedics and related research,* vol. 475, no. 12, pp. 2992-3004. |
| Lyman, S., Lee, Y.Y., Franklin, P.D., Li, W., Cross, M.B. & Padgett, D.E. 2016, "Validation of the KOOS, JR: A Short-form Knee Arthroplasty Outcomes Survey", *Clinical orthopaedics and related research,* vol. 474, no. 6, pp. 1461-1471. |
| Lyman, S., Lee, Y.Y., Franklin, P.D., Li, W., Mayman, D.J. & Padgett, D.E. 2016, "Validation of the HOOS, JR: A Short-form Hip Replacement Survey", *Clinical orthopaedics and related research,* vol. 474, no. 6, pp. 1472-1482. |
| Maempel, J.F., Clement, N.D., Brenkel, I.J. & Walmsley, P.J. 2016, "Range of movement correlates with the Oxford knee score after total knee replacement: A prediction model and validation", *The Knee,* vol. 23, no. 3, pp. 511-516. |
| Maempel, J.F., Clement, N.D., Brenkel, I.J. & Walmsley, P.J. 2015, "Validation of a prediction model that allows direct comparison of the Oxford Knee Score and American Knee Society clinical rating system", *The bone & joint journal,* vol. 97-B, no. 4, pp. 503-509. |
| Mahler, E., Cuperus, N., Bijlsma, J., Vliet Vlieland, T., van den Hoogen, F., den Broeder, A.A. & van den Ende, C.H. 2016, "Responsiveness of four patient-reported outcome measures to assess physical function in patients with knee osteoarthritis", *Scandinavian journal of rheumatology,* vol. 45, no. 6, pp. 518-527. |
| Mahler, E.A., Cuperus, N., Bijlsma, J.W., Vliet Vlieland, T.P., Den Broeder, A.A. & Van Den Ende, C.H. 2015, "Assessment and comparison of responsiveness of four patient reported outcome measures to assess physical function in knee OA: WOMAC-PF subscale responds best", *Annals of the Rheumatic Diseases,* vol. 74, pp. 1185. |
| Maniar, R.N., Maniar, P.R., Chanda, D., Gajbhare, D. & Chouhan, T. 2017, "What is the Responsiveness and Respondent Burden of the New Knee Society Score?", *Clinical orthopaedics and related research,* vol. 475, no. 9, pp. 2218-2227. |
| Matsumoto, M., Baba, T., Homma, Y., Kobayashi, H., Ochi, H., Yuasa, T., Behrend, H. & Kaneko, K. 2015, "Validation study of the Forgotten Joint Score-12 as a universal patient-reported outcome measure", *European Journal of Orthopaedic Surgery and Traumatology,* vol. 25, no. 7, pp. 1141-1145. |
| Matsumoto, M., Baba, T., Ochi, H., Ozaki, Y., Watari, T., Homma, Y. & Kaneko, K. 2017, "Influence of the contralateral hip state after total hip arthroplasty on patient-reported outcomes measured with the Forgotten Joint Score-12", *European Journal of Orthopaedic Surgery and Traumatology,* vol. 27, no. 7, pp. 929-936. |
| Mehta, S.P., Sankar, A., Venkataramanan, V. & Davis, A.M. 2015, "Cross-cultural validation of the ICOAP, physical function short forms of the HOOS, KOOS in patients with hip and knee osteoarthritis", *Physiotherapy (United Kingdom),* vol. 101, pp. eS991-eS992. |
| Morgan, N.L., Driban, J.B., Ransford, G.L., Price, L. & Wang, C. 2013, "Construct validity of promis instruments among patients with symptomatic knee osteoarthritis", *Osteoarthritis and Cartilage,* vol. 21, pp. S139. |
| Parsons, H., Bruce, J., Achten, J., Costa, M.L. & Parsons, N.R. 2014, "Measurement properties of the Disability Rating Index in patients undergoing hip replacement", *Rheumatology (United Kingdom),* vol. 54, no. 1, pp. 64-71. |
| Paulsen, A., Pedersen, A.B., Overgaard, S. & Roos, E.M. 2012, "Feasibility of 4 patient-reported outcome measures in a registry setting", *Acta orthopaedica,* vol. 83, no. 4, pp. 321-327. |
| Peter, W.F., Loos, M., van den Hoek, J. & Terwee, C.B. 2015, "Validation of the Animated Activity Questionnaire (AAQ) for patients with hip and knee osteoarthritis: comparison to home-recorded videos", *Rheumatology international,* vol. 35, no. 8, pp. 1399-1408. |
| Pietrosimone, B., Luc, B.A., Duncan, A., Saliba, S.A., Hart, J.M. & Ingersoll, C.D. 2017, "Association Between the Single Assessment Numeric Evaluation and the Western Ontario and McMaster Universities Osteoarthritis Index", *Journal of athletic training,* vol. 52, no. 6, pp. 526-533. |
| Pollard, B., Johnston, M. & Dixon, D. 2012, "Exploring differential item functioning in the Western Ontario and McMaster Universities Osteoarthritis Index (WOMAC)", *BMC Musculoskeletal Disorders,* vol. 13. |
| Reito, A., Järvistö, A., Jämsen, E., Skyttä, E., Remes, V., Huhtala, H., Niemeläinen, M. & Eskelinen, A. 2017, "Translation and validation of the 12-item Oxford knee score for use in Finland", *BMC Musculoskeletal Disorders,* vol. 18, no. 1, pp. 1-6. |
| Schifferdecker, K.E., Yount, S.E., Kaiser, K., Adachi-Mejia, A., Cella, D., Carluzzo, K.L., Eisenstein, A., Kallen, M.A., Greene, G.J., Eton, D.T. & Fisher, E.S. 2018, "A method to create a standardized generic and condition-specific patient-reported outcome measure for patient care and healthcare improvement", *Quality of Life Research,* vol. 27, no. 2, pp. 367-378. |
| Stegmeier, N., Oak, S.R., O’Rourke, C., Strnad, G., Spindler, K.P., Jones, M., Farrow, L.D., Andrish, J. & Saluan, P. 2017, "No Clinically Significant Difference Between Adult and Pediatric IKDC Subjective Knee Evaluation Scores in Adults", *Sports Health,* vol. 9, no. 5, pp. 450-455. |
| Thienpont, E., Opsomer, G., Koninckx, A. & Houssiau, F. 2014, "Joint awareness in different types of knee arthroplasty evaluated with the forgotten joint score", *Journal of Arthroplasty,* vol. 29, no. 1, pp. 48-51. |
| Thienpont, E., Vanden Berghe, A., Schwab, P.E., Forthomme, J.P. & Cornu, O. 2016, "Joint awareness in osteoarthritis of the hip and knee evaluated with the ‘Forgotten Joint’ Score before and after joint replacement", *Knee Surgery, Sports Traumatology, Arthroscopy,* vol. 24, no. 10, pp. 3346-3351. |
| Unnanuntana, A., Ruangsomboon, P. & Keesukpunt, W. 2018, "Validity and Responsiveness of the Two-Minute Walk Test for Measuring Functional Recovery After Total Knee Arthroplasty", *Journal of Arthroplasty,* . |
| Weel, H., Lindeboom, R., Kuipers, S.E. & Vervest, T.M.J.S. 2017, "Comparison between the Harris- and Oxford Hip Score to evaluate outcomes one-year after total hip arthroplasty", *Acta Orthopaedica Belgica,* vol. 83, no. 1, pp. 98-109. |
| **Clinical effect sudy** |
| Abdel, M.P., Parratte, S., Blanc, G., Ollivier, M., Pomero, V., Viehweger, E. & Argenson, J.-.A. 2014, "No benefit of patient-specific instrumentation in TKA on functional and gait outcomes: A randomized clinical trial", *Clinical orthopaedics and related research,* vol. 472, no. 8, pp. 2468-2476. |
| Abram, S.G., Marsh, A.G., Brydone, A.S., Nicol, F., Mohammed, A. & Spencer, S.J. 2014, "The effect of tibial component sizing on patient reported outcome measures following uncemented total knee replacement", *The Knee,* vol. 21, no. 5, pp. 955-959. |
| Abram, S.G.F., Nicol, F. & Spencer, S.J. 2016, "Patient reported outcomes in three hundred and twenty eight bilateral total knee replacement cases (simultaneous versus staged arthroplasty) using the Oxford Knee Score", *International orthopaedics,* vol. 40, no. 10, pp. 2055-2059. |
| Ageberg, E., Nilsdotter, A., Kosek, E. & Roos, E.M. 2013, "Effects of neuromuscular training (NEMEX-TJR) on patient-reported outcomes and physical function in severe primary hip or knee osteoarthritis: A controlled before-and-after study", *BMC Musculoskeletal Disorders,* vol. 14. |
| Ahearn, N., Metcalfe, A.J., Hassaballa, M.A., Porteous, A.J., Robinson, J.R., Murray, J.R. & Newman, J.H. 2016, "The Journey patellofemoral joint arthroplasty: A minimum 5 year follow-up study", *Knee,* vol. 23, no. 5, pp. 900-904. |
| Ahmed, I., Paraoan, V., Bhatt, D., Mishra, B., Khatri, C., Griffin, D., Metcalfe, A. & Barlow, T. 2018, "Tibial component sizing and alignment of TKR components does not significantly affect patient reported outcome measures at six months. A case series of 474 participants", *International Journal of Surgery,* vol. 52, pp. 67-73. |
| Ali, A., Lindstrand, A., Nilsdotter, A. & Sundberg, M. 2016, "Similar patient-reported outcomes and performance after total knee arthroplasty with or without patellar resurfacing: A randomized study of 74 patients with 6 years of follow-up", *Acta Orthopaedica,* vol. 87, no. 3, pp. 274-279. |
| Altman, R.D., Strand, V., Hochberg, M.C., Gibofsky, A., Markenson, J.A., Hopkins, W.E., Cryer, B., Kivitz, A., Nezzer, J., Imasogie, O. & Young, C.L. 2015, "Low-dose solumatrix diclofenac in the treatment of osteoarthritis: A 1-year, open-label, phase III safety study", *Postgraduate medicine,* vol. 127, no. 5, pp. 517-528. |
| Altuntas, A.O., Alsop, H. & Cobb, J.P. 2013, "Early results of a domed tibia, mobile bearing lateral unicompartmental knee arthroplasty from an independent centre", *Knee,* vol. 20, no. 6, pp. 466-470. |
| Amanatullah, D.F., Meehan, J.P., Cullen, A.B., Kim, S.H. & Jamali, A.A. 2011, "Intermediate-Term Radiographic and Patient Outcomes in Revision Hip Arthroplasty With a Modular Calcar Design and Porous Plasma Coating", *Journal of Arthroplasty,* vol. 26, no. 8, pp. 1451-1454. |
| Anakwe, R.E., Middleton, S.D., Jenkins, P.J., Butler, A.P., Aitken, S.A., Keating, J.F. & Moran, M. 2012, "Total hip replacement in patients with hip fracture: A matched cohort study", *Journal of Trauma and Acute Care Surgery,* vol. 73, no. 3, pp. 738-742. |
| Argenson, J.-., Parratte, S., Ashour, A., Komistek, R.D. & Scuderi, G.R. 2008, "Patient-reported outcome correlates with knee function after a single-design mobile-bearing TKA", *Clinical orthopaedics and related research,* vol. 466, no. 11, pp. 2669-2676. |
| Aro, H., Moritz, N., Lankinen, P., Timlin, S. & Svedström, E. 2011, "A single dose of zoledronic acid prevents periprosthetic bone loss in female patients with cementless total hip arthroplasty", *Journal of Bone and Mineral Research,* vol. 26. |
| Aro, H., Moritz, N., Timlin, S., Lankinen, P. & Svedström, E. 2012, "A single dose of an intravenous bisphosphonate prevents periprosthetic bone loss but does not enhance rsa-evaluated osseointegration of uncemented femoral stems", *HIP International,* vol. 22, no. 4, pp. 443. |
| Artz, N., Dixon, S., Wylde, V., Marques, E., Beswick, A.D., Lenguerrand, E., Blom, A.W. & Gooberman-Hill, R. 2017, "Comparison of group-based outpatient physiotherapy with usual care after total knee replacement: A feasibility study for a randomized controlled trial", *Clinical rehabilitation,* vol. 31, no. 4, pp. 487-499. |
| Bagarić, I., Šarac, H., Borovac, J.A.D., Vlak, T., Bekavac, J. & Hebrang, A. 2014, "Primary total hip arthroplasty: Health related quality of life outcomes", *International orthopaedics,* vol. 38, no. 3, pp. 495-501. |
| Baker, R.K., Kaufman, M.K., Caruthers, E.J., Freisinger, G.M., Lewis, J.M., Schmitt, L.C., Best, T.M., Chaudhari, A.M.W. & Siston, R.A. 2016, "Muscle force & activation patterns during a sit-to-stand transfer in subjects with knee osteoarthritis", *Journal of Orthopaedic Research,* vol. 34. |
| Bastos Filho, R., Magnussen, R.A., Duthon, V., Demey, G., Servien, E., Granjeiro, J.M. & Neyret, P. 2013, "Total knee arthroplasty after high tibial osteotomy: a comparison of opening and closing wedge osteotomy.", *International orthopaedics,* vol. 37, no. 3, pp. 427-431. |
| Beers, L.W., Oldenrijk, J., Scholtes, V.A., Geerdink, C.H., Niers, B.B., Runne, W., Bhandari, M. & Poolman, R.W. 2016, "Curved versus Straight Stem Uncemented Total Hip Arthroplasty Osteoarthritis Multicenter trial (CUSTOM): design of a prospective blinded randomised controlled multicentre trial", *BMJ open,* vol. 6, no. 3. |
| Berend, K.R., Lombardi, A.V., Jr. & Jacobs, C.A. 2017, "The Combination of Preoperative Bone Marrow Lesions and Partial-Thickness Cartilage Loss Did Not Result in Inferior Outcomes After Medial Unicompartmental Knee Arthroplasty", *Journal of Arthroplasty,* vol. 32, no. 10, pp. 3000-3003. |
| Birmingham, T.B., Giffin, J.R., Chesworth, B.M., Bryant, D.M., Litchfield, R.B., Willits, K., Jenkyn, T.R. & Fowler, P.J. 2009, "Medial opening wedge high tibial osteotomy: A prospective cohort study of gait, radiographic, and patient-reported outcomes", *Arthritis Care and Research,* vol. 61, no. 5, pp. 648-657. |
| Birmingham, T.B., Moyer, R., Leitch, K., Chesworth, B., Bryant, D., Willits, K., Litchfield, R., Fowler, P.J. & Giffin, J.R. 2017, "Changes in biomechanical risk factors for knee osteoarthritis and their association with 5-year clinically important improvement after limb realignment surgery", *Osteoarthritis and Cartilage,* vol. 25, no. 12, pp. 1999-2006. |
| Blackburn, J., Lim, D., Harrowell, I., Parry, M.C., Blom, A.W. & Whitehouse, M.R. 2017, "Posterior approach to optimise patient-reported outcome from revision hip arthroplasty", *HIP International,* vol. 27, no. 2, pp. 175-179. |
| Blakey, C.M., Eswaramoorthy, V.K., Hamilton, L.C., Biant, L.C. & Field, R.E. 2009, "Mid-term results of the modular ANCA-Fit femoral component in total hip replacement", *Journal of Bone and Joint Surgery - Series B,* vol. 91, no. 12, pp. 1561-1565. |
| Bolink, S.A.A.N., Grimm, B. & Heyligers, I.C. 2015, "Patient-reported outcome measures versus inertial performance-based outcome measures: A prospective study in patients undergoing primary total knee arthroplasty", *Knee,* vol. 22, no. 6, pp. 618-623. |
| Bolink, S.A.A.N., Lenguerrand, E., Brunton, L.R., Wylde, V., Gooberman-Hill, R., Heyligers, I.C., Blom, A.W. & Grimm, B. 2016, "Assessment of physical function following total hip arthroplasty: Inertial sensor based gait analysis is supplementary to patient-reported outcome measures", *Clinical Biomechanics,* vol. 32, pp. 171-179. |
| Bonnefoy-Mazure, A., Martz, P., Armand, S., Hoffmeyer, P., Sagawa Jr., Y., Suva, D., Turcot, K., Miozzari, H. & Lubbeke, A. 2016, "Knee kinematic recovery one year after total knee arthroplasties: The influence of BMI", *Osteoarthritis and Cartilage,* vol. 24, pp. S101. |
| Boyce, M.B. & Browne, J.P. 2015, "The effectiveness of providing peer benchmarked feedback to hip replacement surgeons based on patientreported outcome measures-results from the PROFILE (Patient-Reported Outcomes: Feedback Interpretation and Learning Experiment) trial: A cluster randomised controlled study", *BMJ Open,* vol. 5, no. 7. |
| Boyle, K.K., Nodzo, S.R., Ferraro, J.T., Augenblick, D.J., Pavlesen, S. & Phillips, M.J. 2018, "Uncemented vs Cemented Cruciate Retaining Total Knee Arthroplasty in Patients With Body Mass Index Greater Than 30", *Journal of Arthroplasty,* vol. 33, no. 4, pp. 1082-1088. |
| Brosky, T., Topp, R., Finley, M., Killian, C., Pariser, D., Brown, K., Bloemer, G. & Stearns, Z. 2011, "Effects of prehabilitation on early rehabilitation outcomes following total knee arthroplasty in patients with knee osteoarthritis", *Physiotherapy (United Kingdom),* vol. 97, pp. eS160. |
| Burn, E., Sanchez-Santos, M.T., Pandit, H.G., Hamilton, T.W., Liddle, A.D., Murray, D.W. & Pinedo-Villanueva, R. 2016, "Ten-year patient-reported outcomes following total and minimally invasive unicompartmental knee arthroplasty: a propensity score-matched cohort analysis", *Knee Surgery, Sports Traumatology, Arthroscopy,* , pp. 1-10. |
| Carey, B.W. & Harty, J. 2018, "A comparison of clinical- and patient-reported outcomes of the cemented ATTUNE and PFC sigma fixed bearing cruciate sacrificing knee systems in patients who underwent total knee replacement with both prostheses in opposite knees", *Journal of Orthopaedic Surgery and Research,* vol. 13, no. 1. |
| Case, R., Jordan, K.P. & Peat, G. 2015, "Prolonged symptom elevation but not progression following incident radiographic knee osteoarthritis: Data from the osteoarthritis initiative", *Annals of the Rheumatic Diseases,* vol. 74, pp. 375-376. |
| Chatterjee, D., McGee, A., Strauss, E., Youm, T. & Jazrawi, L. 2015, "Subchondral Calcium Phosphate is Ineffective for Bone Marrow Edema Lesions in Adults With Advanced Osteoarthritis", *Clinical orthopaedics and related research,* vol. 473, no. 7, pp. 2334-2342. |
| Cherian, J.J., McElroy, M.J., Kapadia, B.H., Bhave, A. & Mont, M.A. 2015, "Prospective case series of NMES for quadriceps weakness and decrease function in patients with osteoarthritis of the knee", *Journal of long-term effects of medical implants,* vol. 25, no. 4, pp. 301-306. |
| Cherian, J.J., Jinnah, A.H., Robinson, K., O'Connor, M.I., Harwin, S.F. & Mont, M.A. 2016, "Prospective, Longitudinal Evaluation of Gender Differences After Total Hip Arthroplasty", *Orthopedics,* vol. 39, no. 2, pp. e391-e396. |
| Choi, N.Y., In, Y., Bae, J.-., Do, J.-., Chung, S.J. & Koh, I.J. 2017, "Are Midterm Patient-Reported Outcome Measures Between Rotating-Platform Mobile-Bearing Prosthesis and Medial-Pivot Prosthesis Different? A Minimum of 5-Year Follow-Up Study", *Journal of Arthroplasty,* vol. 32, no. 3, pp. 824-829. |
| Christen, M., Aghayev, E. & Christen, B. 2014, "Short-term functional versus patient-reported outcome of the bicruciate stabilized total knee arthroplasty: Prospective consecutive case series", *BMC Musculoskeletal Disorders,* vol. 15, no. 1. |
| Clement, N.D., Macdonald, D., Burnett, R., Simpson, A.H.R.W. & Howie, C.R. 2017, "A patient’s perception of their hospital stay influences the functional outcome and satisfaction of total knee arthroplasty", *Archives of orthopaedic and trauma surgery,* vol. 137, no. 5, pp. 693-700. |
| Cole, B.J., Karas, V., Hussey, K., Pilz, K. & Fortier, L.A. 2017, "Hyaluronic Acid Versus Platelet-Rich Plasma", *American Journal of Sports Medicine,* vol. 45, no. 2, pp. 339-346. |
| Collados-Maestre, I., Lizaur-Utrilla, A., Martinez-Mendez, D., Marco-Gomez, L. & Lopez-Prats, F.A. 2016, "Concomitant low back pain impairs outcomes after primary total knee arthroplasty in patients over 65 years: a prospective, matched cohort study", *Archives of orthopaedic and trauma surgery,* vol. 136, no. 12, pp. 1767-1771. |
| Collins, B., Getgood, A., Alomar, A.Z., Giffin, J.R., Willits, K., Fowler, P.J., Birmingham, T.B. & Litchfield, R.B. 2013, "A case series of lateral opening wedge high tibial osteotomy for valgus malalignment", *Knee Surgery, Sports Traumatology, Arthroscopy,* vol. 21, no. 1, pp. 152-160. |
| Collins, J.E., Donnell-Fink, L.A., Yang, H.Y., Usiskin, I.M., Lape, E.C., Wright, J., Katz, J.N. & Losina, E. 2017, "Effect of Obesity on Pain and Functional Recovery Following Total Knee Arthroplasty", *The Journal of bone and joint surgery.American volume,* vol. 99, no. 21, pp. 1812-1818. |
| Conaghan, P., Rannou, F., Arden, N., Everett, S.V., Balshaw, R., Peloso, P.M., Watson, D.J., Sen, S.S. & Taylor, S.D. 2012, "Inadequate pain relief (IPR) in knee osteoarthritis: What does it look like? A European survey of osteoarthritis real world therapies (SORT)", *Osteoarthritis and Cartilage,* vol. 20, pp. S178. |
| Czyzewska, A., Walesiak, K., Krawczak, K., Zukowska, A. & Glinkowski, W.M. 2015, "Effectiveness of preoperative telerehabilitation immediately before total hip replacement for patients suffering severe hip osteoarthritis", *Osteoporosis International,* vol. 26, no. 1, pp. S353-S354. |
| Danford, N., Grosso, M., Heller, M.S., Murtaugh, T., Shah, R.P., Cooper, H.J., Lakra, A. & Geller, J.A. 2017, "Which do patients prefer, unicompartmental or total knee arthroplasty?", *Journal of Clinical Orthopaedics and Trauma,* . |
| Degen, R.M., Matz, J., Teeter, M.G., Lanting, B.A., Howard, J.L. & Mccalden, R.W. 2017, "Does Posterior Condylar Offset Affect Clinical Results following Total Knee Arthroplasty?", *Journal of Knee Surgery,* . |
| Dettmer, M., Pourmoghaddam, A. & Kreuzer, S.W. 2015, "Comparison of Patient-Reported Outcome from Neck-Preserving, Short-Stem Arthroplasty and Resurfacing Arthroplasty in Younger Osteoarthritis Patients", *Advances in Orthopedics,* vol. 2015. |
| Dixon, S., Blom, A.W., Whitehouse, M.R. & Wylde, V. 2014, "Comparison of patient reported outcomes after Triathlon® and Kinemax Plus prostheses", *Annals of the Royal College of Surgeons of England,* vol. 96, no. 1, pp. 61-66. |
| Dowsey, M.M., Nikpour, M., Dieppe, P. & Choong, P.F.M. 2012, "Associations between pre-operative radiographic changes and outcomes after total knee joint replacement for osteoarthritis", *Osteoarthritis and Cartilage,* vol. 20, no. 10, pp. 1095-1102. |
| Dowsey, M.M., Robertsson, O., Sundberg, M., Lohmander, L.S., Choong, P.F.M. & W-Dahl, A. 2017, "Variations in pain and function before and after total knee arthroplasty: a comparison between Swedish and Australian cohorts", *Osteoarthritis and Cartilage,* vol. 25, no. 6, pp. 885-891. |
| Eneqvist, T., Nemes, S., Brisby, H., Fritzell, P., Garellick, G. & Rolfson, O. 2017, "Lumbar surgery prior to total hip arthroplasty is associated with worse patient-reported outcomes", *The bone & joint journal,* vol. 99-B, no. 6, pp. 759-765. |
| Eyvazov, K., Eyvazov, B., Basar, S., Nasto, L.A. & Kanatli, U. 2016, "Effects of total hip arthroplasty on spinal sagittal alignment and static balance: a prospective study on 28 patients", *European Spine Journal,* vol. 25, no. 11, pp. 3615-3621. |
| Forster-Horváth, C., Artz, N., Hassaballa, M.A., Robinson, J.R., Porteous, A.J., Murray, J.R. & Newman, J.H. 2016, "Survivorship and clinical outcome of the minimally invasive Uniglide medial fixed bearing, all-polyethylene tibia, unicompartmental knee arthroplasty at a mean follow-up of 7.3 years", *Knee,* vol. 23, no. 6, pp. 981-986. |
| Freisinger, G.M., Hutter, E.E., Lewis, J., Granger, J.F., Glassman, A.H., Beal, M.D., Pan, X., Schmitt, L.C., Siston, R.A. & Chaudhari, A.M.W. 2017, "Relationships between varus–valgus laxity of the severely osteoarthritic knee and gait, instability, clinical performance, and function", *Journal of Orthopaedic Research,* vol. 35, no. 8, pp. 1644-1652. |
| Fuentes, A., Bureau, N.J., Boivin, K., Mezghani, N., Ouakrim, Y., De Guise, J.A. & Hagemeister, N. 2013, "Knee frontal plane dynamic alignment in knee osteoarthritis patients: Impact of a 12-week physical therapy program and relationship with functional scores", *PM and R,* vol. 5, no. 9, pp. S207-S208. |
| Gallo, M.C., Samaan, M.A., Pedoia, V., Souza, R.B. & Majumdar, S. 2017, "A 3-year longitudinal study in hip osteoarthritis: Association between baseline biomechanics and changes in patient reported outcomes and cartilage compositional T1ρ and T2", *Journal of Orthopaedic Research,* vol. 35. |
| Getgood, A., Collins, B., Slynarski, K., Kurowska, E., Parker, D., Engebretsen, L., MacDonald, P.B. & Litchfield, R. 2013, "Short-term safety and efficacy of a novel high tibial osteotomy system: A case controlled study", *Knee Surgery, Sports Traumatology, Arthroscopy,* vol. 21, no. 1, pp. 260-269. |
| Goh, G.S.-., Liow, M.H.L., Bin Abd Razak, H.R., Tay, D.K.-., Lo, N.-. & Yeo, S.-. 2017, "Patient-Reported Outcomes, Quality of Life, and Satisfaction Rates in Young Patients Aged 50 Years or Younger After Total Knee Arthroplasty", *Journal of Arthroplasty,* vol. 32, no. 2, pp. 419-425. |
| Gordon, M., Paulsen, A., Overgaard, S., Garellick, G., Pedersen, A.B. & Rolfson, O. 2013, "Factors influencing health-related quality of life after total hip replacement - A comparison of data from the Swedish and Danish hip arthroplasty registers", *BMC Musculoskeletal Disorders,* vol. 14. |
| Gray, B.L., Stambough, J.B., Baca, G.R., Schoenecker, P.L. & Clohisy, J.C. 2015, "Comparison of contemporary periacetabular osteotomy for hip dysplasia with total hip arthroplasty for hip osteoarthritis", *Bone and Joint Journal,* vol. 97-B, no. 10, pp. 1322-1327. |
| Greene, M.E., Rolfson, O., Gordon, M., Annerbrink, K., Malchau, H. & Garellick, G. 2016, "Is the use of antidepressants associated with patient-reported outcomes following total hip replacement surgery?", *Acta Orthopaedica,* vol. 87, no. 5, pp. 444-451. |
| Gromov, K., Greene, M.E., Sillesen, N.H., Troelsen, A., Malchau, H., Huddleston, J.I., Emerson, R., Garcia-Cimbrelo, E., Gebuhr, P. & Multicenter Writing Committee 2014, "Regional differences between US and Europe in radiological osteoarthritis and self assessed quality of life in patients undergoing total hip arthroplasty surgery", *The Journal of arthroplasty,* vol. 29, no. 11, pp. 2078-2083. |
| Gwynne-Jones, D. & Iosua, E. 2016, "Rationing of hip and knee replacement: Effect on the severity of patient-reported symptoms and the demand for surgery in Otago", *New Zealand Medical Journal,* vol. 129, no. 1432, pp. 59-66. |
| Gwynne-Jones, D.P., Iosua, E.E. & Stout, K.M. 2016, "Rationing for Total Hip and Knee Arthroplasty Using the New Zealand Orthopaedic Association Score: Effectiveness and Comparison With Patient-Reported Scores", *Journal of Arthroplasty,* vol. 31, no. 5, pp. 957-962. |
| Haase, E., Kopkow, C., Beyer, F., Lützner, J., Kirschner, S., Hartmann, A., Schmitt, J. & Günther, K.-. 2016, "Patient-reported outcomes and outcome predictors after primary total hip arthroplasty: Results from the Dresden hip surgery registry", *HIP International,* vol. 26, no. 1, pp. 73-81. |
| Hall, D.P., Srikantharajah, D., Anakwe, R.E., Gaston, P. & Howie, C.R. 2009, "Patient-reported outcome following metal-on-metal resurfacing of the hip and total hip replacement", *HIP International,* vol. 19, no. 3, pp. 245-250. |
| Harwin, S.F., Issa, K., Given, K., Hitt, K.D., Greene, K.A., Pivec, R., Kester, M. & Mont, M.A. 2013, "Clinical and patient-reported outcomes of primary TKA with a single-radius design", *Orthopedics,* vol. 36, no. 7, pp. e877; e882. |
| Harwin, S.F., Patel, N.K., Chughtai, M., Khlopas, A., Ramkumar, P.N., Roche, M. & Mont, M.A. 2017, "Outcomes of Newer Generation Cementless Total Knee Arthroplasty: Beaded Periapatite-Coated vs Highly Porous Titanium-Coated Implants", *Journal of Arthroplasty,* vol. 32, no. 7, pp. 2156-2160. |
| Haynes, J., Sassoon, A., Nam, D., Schultz, L. & Keeney, J. 2017, "Younger patients have less severe radiographic disease and lower reported outcome scores than older patients undergoing total knee arthroplasty", *Knee,* vol. 24, no. 3, pp. 663-669. |
| Henderson, F., Smith, I., Baird, K., Holt, G. & Khan, A. 2017, "A single surgeon's experience of metal-on-metal hip resurfacing arthroplasty in a district general hospital: 9-year clinical results using the Conserve Plus resurfacing system", *Current Orthopaedic Practice,* vol. 28, no. 6, pp. 557-562. |
| Hermann, A., Holsgaard-Larsen, A., Zerahn, B., Mejdahl, S. & Overgaard, S. 2016, "Preoperative progressive explosive-type resistance training is feasible and effective in patients with hip osteoarthritis scheduled for total hip arthroplasty - a randomized controlled trial", *Osteoarthritis and Cartilage,* vol. 24, no. 1, pp. 91-98. |
| Hiyama, Y., Wada, O., Nakakita, S. & Mizuno, K. 2016, "Joint awareness after total knee arthroplasty is affected by pain and quadriceps strength", *Orthopaedics and Traumatology: Surgery and Research,* vol. 102, no. 4, pp. 435-439. |
| Holsgaard-Larsen, A., Jensen, C., Overgaard, S., Rosenlund, S. & Broeng, L. 2017, "Patient-reported outcome after total hip arthroplasty: comparison between lateral and posterior approach", *Acta Orthopaedica,* vol. 88, no. 3, pp. 239-247. |
| Hsu, W.-., Hsu, W.-., Shen, W.-., Lin, Z.-., Chang, S.-. & Hsu, R.W.-. 2017, "Circuit training enhances function in patients undergoing total knee arthroplasty: A retrospective cohort study", *Journal of Orthopaedic Surgery and Research,* vol. 12, no. 1. |
| Hussey, K., Cole, B.J., Pilz, K., Karas, V. & Fortier, L.A. 2017, "Hyaluronic Acid Versus Platelet-Rich Plasma", *American Journal of Sports Medicine,* vol. 45, no. 2, pp. 339-346. |
| Indelli, P.F., Pipino, G., Johnson, P., Graceffa, A. & Marcucci, M. 2016, "Posterior-stabilized total knee arthroplasty: a matched pair analysis of a classic and its evolutional design", *Arthroplasty Today,* vol. 2, no. 4, pp. 193-198. |
| Ismailidis, P., Kuster, M.S., Jost, B., Giesinger, K. & Behrend, H. 2017, "Clinical outcome of increased flexion gap after total knee arthroplasty. Can controlled gap imbalance improve knee flexion?", *Knee surgery, sports traumatology, arthroscopy : official journal of the ESSKA,* vol. 25, no. 6, pp. 1705-1711. |
| Issa, K., Harwin, S.F., Malkani, A.L., Bonutti, P.M., Scillia, A. & Mont, M.A. 2016, "Bariatric orthopaedics: Total hip arthroplasty in super-obese patients (those with a BMI of ≥50 kg/m2)", *Journal of Bone and Joint Surgery - American Volume,* vol. 98, no. 3, pp. 180-185. |
| Issa, K., Pierce, T.P., Harwin, S.F., Scillia, A.J., Festa, A. & Mont, M.A. 2017, "No Decrease in Knee Survivorship or Outcomes Scores for Patients With HIV Infection Who Undergo TKA", *Clinical orthopaedics and related research,* vol. 475, no. 2, pp. 465-471. |
| Jacobs, C.A., Christensen, C.P. & Karthikeyan, T. 2016, "Greater medial compartment forces during TKA associated with improved patient satisfaction and function", *Journal of Orthopaedic Research,* vol. 34. |
| Jacobs, C.A., Christensen, C.P. & Karthikeyan, T. 2016, "Greater Medial Compartment Forces During Total Knee Arthroplasty Associated With Improved Patient Satisfaction and Ability to Navigate Stairs", *Journal of Arthroplasty,* vol. 31, no. 9, pp. 87-90. |
| Jacobson, A.F., Umberger, W.A., Palmieri, P.A., Alexander, T.S., Myerscough, R.P., Draucker, C.B., Steudte-Schmiedgen, S. & Kirschbaum, C. 2016, "Guided imagery for total knee replacement: A randomized, placebo-controlled pilot study", *Journal of Alternative and Complementary Medicine,* vol. 22, no. 7, pp. 563-575. |
| Jameson, S.S., Mason, J., Baker, P., Gregg, P.J., McMurtry, I.A., Deehan, D.J. & Reed, M.R. 2014, "A comparison of surgical approaches for primary hip arthroplasty: a cohort study of patient reported outcome measures (PROMs) and early revision using linked national databases", *The Journal of arthroplasty,* vol. 29, no. 6, pp. 1248-1255.e1. |
| Jameson, S.S., Mason, J., Baker, P., Gregg, P.J., Porter, M., Deehan, D.J. & Reed, M.R. 2015, "Have cementless and resurfacing components improved the medium-term results of hip replacement for patients under 60 years of age?", *Acta orthopaedica,* vol. 86, no. 1, pp. 7-17. |
| Jameson, S.S., Mason, J., Baker, P.N., Gregg, P.J., Deehan, D.J. & Reed, M.R. 2015, "Implant Optimisation for Primary Hip Replacement in Patients over 60 Years with Osteoarthritis: A Cohort Study of Clinical Outcomes and Implant Costs Using Data from England and Wales", *PloS one,* vol. 10, no. 11, pp. e0140309. |
| Jameson, S.S., Mason, J.M., Baker, P.N., Gregg, P.J., Deehan, D.J. & Reed, M.R. 2015, "No functional benefit of larger femoral heads and alternative bearings at 6 months following primary hip replacement", *Acta orthopaedica,* vol. 86, no. 1, pp. 32-40. |
| Jerger, K., Juberg, M., Allen, K.D., Dmitrieva, N.O., Keever, T. & Perlman, A.I. 2015, "Massage for knee osteoarthritis", *Integrative Medicine Research,* vol. 4, no. 1, pp. 104. |
| Jonbergen, H.P., Scholtes, V.A., Kampen, A. & Poolman, R.W. 2011, "A randomised, controlled trial of circumpatellar electrocautery in total knee replacement without patellar resurfacing", *Journal of bone and joint surgery.British volume,* vol. 93, no. 8, pp. 1054-1059. |
| Jones, G.G., Kotti, M., Wiik, A.V., Collins, R., Brevadt, M.J., Strachan, R.K. & Cobb, J.P. 2016, "Gait comparison of unicompartmental and total knee arthroplasties with healthy controls", *The bone & joint journal,* vol. 98-B, no. 10 Supple B, pp. 16-21. |
| Juberg, M., Jerger, K.K., Allen, K.D., Dmitrieva, N.O., Keever, T. & Perlman, A.I. 2015, "Pilot study of massage in Veterans with knee osteoarthritis", *Journal of Alternative and Complementary Medicine,* vol. 21, no. 6, pp. 333-338. |
| Judge, A., Arden, N.K., Kiran, A., Price, A., Javaid, M.K., Beard, D., Murray, D. & Field, R.E. 2012, "Interpretation of patient-reported outcomes for hip and knee replacement surgery: Identification of thresholds associated with satisfaction with surgery", *Journal of Bone and Joint Surgery - Series B,* vol. 94 B, no. 3, pp. 412-418. |
| Judge, A., Cooper, C., Williams, S., Dreinhoefer, K. & Dieppe, P. 2010, "Patient-reported outcomes one year after primary hip replacement in a European collaborative cohort", *Arthritis Care and Research,* vol. 62, no. 4, pp. 480-488. |
| Kahn, T.L. & Schwarzkopf, R. 2015, "Does Total Knee Arthroplasty Affect Physical Activity Levels? Data from the Osteoarthritis Initiative", *Journal of Arthroplasty,* vol. 30, no. 9, pp. 1521-1525. |
| Kawakami, Y., Matsumoto, T., Takayama, K., Ishida, K., Nakano, N., Matsushita, T., Kuroda, Y., Patel, K., Kuroda, R. & Kurosaka, M. 2015, "Intermediate-term comparison of posterior cruciate-retaining versus posterior-stabilized total knee arthroplasty using the new knee scoring system", *Orthopedics,* vol. 38, no. 12, pp. e1127-e1132. |
| Kearey, P., Popple, A.E., Warren, J., Davis, T., Bellamy, N., Barui, E., Burne, S., Cooke, R., Craig, M., Dalton, P., Dekkers, M., Edelman, J., Fine, S., Gamboa, G., Hall, S., Ibrahim, A., Inderjeeth, C., Lim, K., Mazur, M., Morgan, D.A.F., Myers, P., Nicoll, A., Nicholls, D., Paterson, R., Shanahan, M., Stockman, A., Watson, S. & for the LOBRAS Study Group 2017, "Improvement in condition-specific and generic quality of life outcomes in patients with knee osteoarthritis following single-injection Synvisc: results from the LOBRAS study", *Current medical research and opinion,* vol. 33, no. 3, pp. 409-419. |
| Kim, M.S., Koh, I.J., Choi, Y.J., Lee, J.Y. & In, Y. 2017, "Differences in Patient-Reported Outcomes Between Unicompartmental and Total Knee Arthroplasties: A Propensity Score-Matched Analysis", *Journal of Arthroplasty,* vol. 32, no. 5, pp. 1453-1459. |
| Kolisek, F.R., Chughtai, M., Mistry, J.B., Elmallah, R.K., Jaggard, C., Malkani, A.L., Masini, M.A., Harwin, S.F. & Mont, M.A. 2016, "Outcomes of Second-Generation Tapered Wedge Femoral Stem", *Surgical technology international,* vol. 28, pp. 275-279. |
| Kon, E., Engebretsen, L., Verdonk, P., Nehrer, S. & Filardo, G. 2018, "Clinical Outcomes of Knee Osteoarthritis Treated With an Autologous Protein Solution Injection: A 1-Year Pilot Double-Blinded Randomized Controlled Trial", *American Journal of Sports Medicine,* vol. 46, no. 1, pp. 171-180. |
| Kulshrestha, V., Datta, B., Kumar, S. & Mittal, G. 2017, "Outcome of Unicondylar Knee Arthroplasty vs Total Knee Arthroplasty for Early Medial Compartment Arthritis: A Randomized Study", *Journal of Arthroplasty,* vol. 32, no. 5, pp. 1460-1469. |
| Kumar, D., Wise, B., Majumdar, S., Souza, R.B. & Lane, N.E. 2015, "Vibratory deficits in individuals with and without mildmoderate radiographic hip osteoarthritis", *Osteoarthritis and Cartilage,* vol. 23, pp. A333-A334. |
| Laires, P., Laíns, J., Miranda, L., Cernadas, R., Pereira Da Silva, J., Gomes, J.M., Peloso, P.M., Taylor, S.D. & Silva, J.C. 2014, "Inadequate pain relief among patients with primary knee osteoarthritis-analysis from the portuguese sample of the survey of osteoarthritis real world therapies (SORT)", *Value in Health,* vol. 17, no. 7, pp. A386. |
| Laires, P.A., Laíns, J., Miranda, L.C., Cernadas, R., Rajagopalan, S., Taylor, S.D. & Silva, J.C. 2017, "Inadequate pain relief among patients with primary knee osteoarthritis", *Revista Brasileira de Reumatologia,* vol. 57, no. 3, pp. 229-237. |
| Larsen, K., Hansen, T.B., Søballe, K. & Kehlet, H. 2010, "Patient-reported outcome after fast-track hip arthroplasty: A prospective cohort study", *Health and Quality of Life Outcomes,* vol. 8. |
| Lavie, L.G., Parsons, M., Lensing, G., Leonardi, C. & Dasa, V. 2017, "Is outpatient total knee arthroplasty safe and effective?", *Journal of Orthopaedic Research,* vol. 35. |
| Lehnen, K., Giesinger, K., Warschkow, R., Porter, M., Koch, E. & Kuster, M.S. 2011, "Clinical outcome using a ligament referencing technique in CAS versus conventional technique", *Knee Surgery, Sports Traumatology, Arthroscopy,* vol. 19, no. 6, pp. 887-892. |
| Leta, T.H., Lygre, S.H., Skredderstuen, A., Hallan, G., Gjertsen, J.E., Rokne, B. & Furnes, O. 2016, "Outcomes of Unicompartmental Knee Arthroplasty After Aseptic Revision to Total Knee Arthroplasty: A Comparative Study of 768 TKAs and 578 UKAs Revised to TKAs from the Norwegian Arthroplasty Register (1994 to 2011)", *The Journal of bone and joint surgery.American volume,* vol. 98, no. 6, pp. 431-440. |
| Liddle, A.D., Judge, A., Pandit, H. & Murray, D.W. 2014, "Determinants of revision and functional outcome following unicompartmental knee replacement", *Osteoarthritis and cartilage,* vol. 22, no. 9, pp. 1241-1250. |
| Liddle, A.D., Pandit, H., Judge, A. & Murray, D.W. 2015, "Patient-reported outcomes after total and unicompartmental knee arthroplasty: a study of 14,076 matched patients from the National Joint Registry for England and Wales", *The bone & joint journal,* vol. 97-B, no. 6, pp. 793-801. |
| Lim, J.B., Chou, A.C., Chong, H.C., Lo, N.N., Chia, S., Tay, K.J. & Yeo, S.J. 2015, "Are patients more satisfied and have better functional outcome after bilateral total knee arthroplasty as compared to total hip arthroplasty and unilateral total knee arthroplasty surgery? A two-year follow-up study", *Acta Orthopaedica Belgica,* vol. 81, no. 4, pp. 682-689. |
| Lim, J.B.T., Chong, H.C., Pang, H.N., Tay, K.J.D., Chia, S.L., Lo, N.N. & Yeo, S.J. 2017, "Revision total knee arthroplasty for failed high tibial osteotomy and unicompartmental knee arthroplasty have similar patient-reported outcome measures in a two-year follow-up study", *Bone and Joint Journal,* vol. 99B, no. 10, pp. 1329-1334. |
| Lindgren, J.V., Wretenberg, P., Karrholm, J., Garellick, G. & Rolfson, O. 2014, "Patient-reported outcome is influenced by surgical approach in total hip replacement: a study of the Swedish Hip Arthroplasty Register including 42,233 patients", *The bone & joint journal,* vol. 96-B, no. 5, pp. 590-596. |
| Lingard, E.A., Muthumayandi, K. & Holland, J.P. 2009, "Comparison of patient-reported outcomes between hip resurfacing and total hip replacement", *Journal of Bone & Joint Surgery, British Volume,* vol. 91, no. 12, pp. 1550-1554. |
| Liu, S.-., Driban, J.B., Eaton, C.B., McAlindon, T.E., Harrold, L.R. & Lapane, K.L. 2016, "Objectively Measured Physical Activity and Symptoms Change in Knee Osteoarthritis", *American Journal of Medicine,* vol. 129, no. 5, pp. 497-505.e1. |
| Lostak, J., Gallo, J. & Zapletalova, J. 2016, "Patient Satisfaction after Total Knee Arthroplasty. Analysis of Pre-Operative and Peri-Operative Parameters Influencing Results in 826 Patients", *Acta Chirurgiae Orthopaedicae et Traumatologiae Cechoslovaca,* vol. 83, no. 2, pp. 94-101. |
| Luna, I.E., Kehlet, H., Peterson, B., Wede, H.R., Hoevsgaard, S.J. & Aasvang, E.K. 2017, "Early patient-reported outcomes versus objective function after total hip and knee arthroplasty", *Bone and Joint Journal,* vol. 99B, no. 9, pp. 1167-1175. |
| Malviya, A., Ramaskandhan, J.R., Bowman, R., Kometa, S., Hashmi, M., Lingard, E. & Holland, J.P. 2011, "What advantage is there to be gained using large modular metal-on-metal bearings in routine primary hip replacement? A preliminary report of a prospective randomised controlled trial", *Journal of Bone and Joint Surgery - Series B,* vol. 93 B, no. 12, pp. 1602-1609. |
| Martin, A., Quah, C., Syme, G., Lammin, K., Segaren, N. & Pickering, S. 2015, "Long term survivorship following Scorpio Total Knee Replacement", *The Knee,* vol. 22, no. 3, pp. 192-196. |
| Matsumoto, K., Ogawa, H., Yoshioka, H. & Akiyama, H. 2017, "Postoperative Anteroposterior Laxity Influences Subjective Outcome After Total Knee Arthroplasty", *Journal of Arthroplasty,* vol. 32, no. 6, pp. 1845-1849. |
| McDonough, C.M., Stoiber, E., Tomek, I.M., Ni, P., Kim, Y.-., Tian, F. & Jette, A.M. 2016, "Sensitivity to change of a computer adaptive testing instrument for outcome measurement after hip and knee arthroplasty and periacetabular osteotomy", *Journal of Orthopaedic and Sports Physical Therapy,* vol. 46, no. 9, pp. 756-767. |
| Meyer, M.A., Leroux, T.S., Levy, D.M., Tilton, A.K., Lewis, P.B., Yanke, A.B. & Cole, B.J. 2017, "Flexion posteroanterior radiographs affect both enrollment for and outcomes after injection therapy for knee osteoarthritis", *Orthopaedic Journal of Sports Medicine,* vol. 5, no. 5. |
| Meyer, M.A., Leroux, T.S., Levy, D.M., Tilton, A.K., Lewis, P.B., Yanke, A.B. & Cole, B.J. 2016, "Radiograph view influences patient selection and patient-reported outcomes in injection therapy for knee osteoarthritis", *Osteoarthritis and Cartilage,* vol. 24, pp. S291-S292. |
| Middleton, S.W.F., Toms, A.D., Schranz, P.J. & Mandalia, V.I. 2018, "Mid-term survivorship and clinical outcomes of the Avon patellofemoral joint replacement", *Knee,* vol. 25, no. 2, pp. 323-328. |
| Mikkelsen, L.R., Petersen, M.K., Sï¿½balle, K., Mikkelsen, S. & Mechlenburg, I. 2014, "Does reduced movement restrictions and use of assistive devices affect rehabilitation outcome after total hip replacement? A non-randomized, controlled study", *European Journal of Physical and Rehabilitation Medicine,* vol. 50, no. 4, pp. 383-393. |
| Mistry, J.B., Elmallah, R.K., Chughtai, M., Oktem, M., Harwin, S.F. & Mont, M.A. 2016, "Long-Term Survivorship and Clinical Outcomes of a Single Radius Total Knee Arthroplasty", *Surgical technology international,* vol. 28, pp. 247-251. |
| Moore, K., Crossley, K.M., Fransen, M., Russell, T. & Kemp, J.L. 2015, "A feasibility trial for the efficacy of physiotherapy intervention for early-onset hip osteoarthritis", *Osteoarthritis and Cartilage,* vol. 23, pp. A371-A372. |
| Morrison, R.J.M., Bunn, D., Gray, W.K., Baker, P.N., White, C., Rangan, A., Rankin, K.S. & Reed, M.R. 2017, "VASO (Vitamin D and Arthroplasty Surgery Outcomes) study - supplementation of vitamin D deficiency to improve outcomes after total hip or knee replacement: Study protocol for a randomised controlled feasibility trial", *Trials,* vol. 18, no. 1. |
| Moyer, R., Birmingham, T., Boulougouris, A. & Giffin, J.R. 2016, "Patient-reported outcomes after a 12-week non-operative program differ between patients preparing to undergo surgery compared to patients that are not", *Osteoarthritis and Cartilage,* vol. 24, pp. S496-S497. |
| Moyer, R., Birmingham, T., Pinto, R., Leitch, K., Chesworth, B. & Giffin, J.R. 2016, "Changes in gait biomechanics are associated with longterm clinically important improvements in patientreported outcomes", *Osteoarthritis and Cartilage,* vol. 24, pp. S27. |
| Murakami, K., Hamai, S., Okazaki, K., Ikebe, S., Nakahara, H., Higaki, H., Shimoto, T., Mizu-uchi, H., Kuwashima, U. & Iwamoto, Y. 2017, "Kinematic analysis of stair climbing in rotating platform cruciate-retaining and posterior-stabilized mobile-bearing total knee arthroplasties", *Archives of orthopaedic and trauma surgery,* vol. 137, no. 5, pp. 701-711. |
| Naal, F.D., Impellizzeri, F.M., Lenze, U., Wellauer, V., von Eisenhart-Rothe, R. & Leunig, M. 2015, "Clinical improvement and satisfaction after total joint replacement: a prospective 12-month evaluation on the patients’ perspective", *Quality of Life Research,* vol. 24, no. 12, pp. 2917-2925. |
| Naili, J.E., Iversen, M.D., Esbjörnsson, A.-., Hedström, M., Schwartz, M.H., Häger, C.K. & Broström, E.W. 2017, "Deficits in functional performance and gait one year after total knee arthroplasty despite improved self-reported function", *Knee Surgery, Sports Traumatology, Arthroscopy,* vol. 25, no. 11, pp. 3378-3386. |
| Naylor, J.M., Kamalasena, G., Hayen, A., Harris, I.A. & Adie, S. 2013, "Can the oxford scores be used to monitor symptomatic progression of patients awaiting knee or hip arthroplasty?", *Journal of Arthroplasty,* vol. 28, no. 9, pp. 1454-1458. |
| Nebergall, A.K., Troelsen, A., Rubash, H.E., Malchau, H., Rolfson, O. & Greene, M.E. 2016, "Five-Year Experience of Vitamin E-Diffused Highly Cross-Linked Polyethylene Wear in Total Hip Arthroplasty Assessed by Radiostereometric Analysis", *Journal of Arthroplasty,* vol. 31, no. 6, pp. 1251-1255. |
| Nerhus, T.K., Ekeland, A., Solberg, G., Olsen, B.H., Madsen, J.E. & Heir, S. 2017, "No difference in time-dependent improvement in functional outcome following closing wedge versus opening wedge high tibial osteotomy", *Bone and Joint Journal,* vol. 99B, no. 9, pp. 1157-1166. |
| Nguyen, L.-.L., Sing, D.C. & Bozic, K.J. 2016, "Preoperative Reduction of Opioid Use Before Total Joint Arthroplasty", *Journal of Arthroplasty,* vol. 31, no. 9, pp. 282-287. |
| Nielsen, K.A., Thomsen, M.G., Latifi, R., Kallemose, T., Husted, H. & Troelsen, A. 2016, "Does post-operative knee awareness differ between knees in bilateral simultaneous total knee arthroplasty? Predictors of high or low knee awareness", *Knee Surgery, Sports Traumatology, Arthroscopy,* vol. 24, no. 10, pp. 3352-3358. |
| Nijman, T.H., Scholtes, V.A., De Meulemeester, F.R.A.J., Van Der Hart, C.P. & Poolman, R.W. 2013, "Short-term functional outcome after hip resurfacing surgery", *European Journal of Orthopaedic Surgery and Traumatology,* vol. 23, no. 2, pp. 197-202. |
| Oiestad, B.E., Osteras, N., Frobell, R., Grotle, M., Brogger, H. & Risberg, M.A. 2013, "Efficacy of strength and aerobic exercise on patient-reported outcomes and structural changes in patients with knee osteoarthritis: study protocol for a randomized controlled trial", *BMC musculoskeletal disorders,* vol. 14. |
| Øiestad, B.E., Østerås, N., Frobell, R., Grotle, M., Brøgger, H. & Risberg, M.A. 2013, "Efficacy of strength and aerobic exercise on patient-reported outcomes and structural changes in patients with knee osteoarthritis: Study protocol for a randomized controlled trial", *BMC Musculoskeletal Disorders,* vol. 14. |
| O'Leary, H., Smart, K., Moloney, N. & Doody, C. 2015, "Clinical measures of pain sensitization in moderate to severe knee osteoarthritis", *Physiotherapy (United Kingdom),* vol. 101, pp. eS1136-eS1137. |
| Ollivier, M., Parratte, S., Lunebourg, A., Viehweger, E. & Argenson, J.-. 2016, "The John Insall Award: No Functional Benefit After Unicompartmental Knee Arthroplasty Performed With Patient-specific Instrumentation: A Randomized Trial", *Clinical orthopaedics and related research,* vol. 474, no. 1, pp. 60-68. |
| Parratte, S., Ollivier, M., Lunebourg, A., Flecher, X. & Argenson, J.-.A. 2016, "No Benefit After THA Performed With Computer-assisted Cup Placement: 10-year Results of a Randomized Controlled Study", *Clinical orthopaedics and related research,* vol. 474, no. 10, pp. 2085-2093. |
| Parratte, S., Ollivier, M., Lunebourg, A., Verdier, N. & Argenson, J.N. 2017, "Do Stemmed Tibial Components in Total Knee Arthroplasty Improve Outcomes in Patients With Obesity?", *Clinical orthopaedics and related research,* vol. 475, no. 1, pp. 137-145. |
| Parry, M.C., Vioreanu, M.H., Garbuz, D.S., Masri, B.A. & Duncan, C.P. 2016, "The Wagner Cone Stem for the Management of the Challenging Femur in Primary Hip Arthroplasty", *Journal of Arthroplasty,* vol. 31, no. 8, pp. 1767-1772. |
| Peloso, P.M., Moore, R.A., Chen, W.-., Lin, H.-., Gates, D.F., Straus, W.L. & Popmihajlov, Z. 2016, "Osteoarthritis patients with pain improvement are highly likely to also have improved quality of life and functioning. A post hoc analysis of a clinical trial", *Scandinavian Journal of Pain,* vol. 13, pp. 175-181. |
| Perrone, F.L., Baron, S., Suero, E.M., Lausmann, C., Kendoff, D., Zahar, A., Gehrke, T. & Citak, M. 2018, "Patient-reported outcome measures (PROMs) in patients undergoing patellofemoral arthroplasty and total knee replacement: A comparative study", *Technology and health care : official journal of the European Society for Engineering and Medicine,* . |
| Perruccio, A.V., Badley, E.M., Hogg-Johnson, S. & Davis, A. 2009, "The significance of self-rated health and mental well-being in predicting outcomes following TJR surgery for OA", *Arthritis and Rheumatism,* vol. 60, pp. 1392. |
| Perruccio, A.V., Gandhi, R., Badley, E.M., Mahomed, N.N., Power, J.D. & Davis, A.M. 2014, "The negative influence of multiple symptomatic joint involvement on patient-reported outcomes following TJR for OA can last up to 5 years", *Osteoarthritis and Cartilage,* vol. 22, pp. S181. |
| Postler, A.E., Beyer, F., Wegner, T., Lützner, J., Hartmann, A., Ojodu, I. & Günther, K.-. 2017, "Patient-reported outcomes after revision surgery compared to primary total hip arthroplasty", *HIP International,* vol. 27, no. 2, pp. 180-186. |
| Primeau, C.A., Solomon, S.T., Birmingham, T.B., Moyer, R.F., Leitch, K.M. & Giffin, J.R. 2016, "Effect of congenital vs. acquired varus on patientreported outcomes after high tibial osteotomy", *Osteoarthritis and Cartilage,* vol. 24, pp. S513. |
| Rady, A.E., Asal, M.K. & Bassiony, A.A. 2010, "The use of a constrained cementless acetabular component for instability in total hip replacement", *HIP International,* vol. 20, no. 4, pp. 434-439. |
| Ramaesh, R., Jenkins, P., Lane, J.V., Knight, S., Macdonald, D. & Howie, C. 2014, "Personality, function and satisfaction in patients undergoing total hip or knee replacement", *Journal of Orthopaedic Science,* vol. 19, no. 2, pp. 275-281. |
| Riis, R.G., Henriksen, M., Klokker, L., Bartholdy, C., Ellegaard, K., Bandak, E., Hansen, B.B., Bliddal, H. & Boesen, M. 2016, "The effects of intra-articular glucocorticoids and exercise on pain and synovitis assessed on static and dynamic magnetic resonance imaging in knee osteoarthritis: exploratory outcomes from a randomized controlled trial", *Osteoarthritis and cartilage.(no pagination), 2016,* vol. Date of Publication: April 14. |
| Riis, R.G.C., Henriksen, M., Klokker, L., Bartholdy, C., Ellegaard, K., Bandak, E., Hansen, B.B., Bliddal, H. & Boesen, M. 2017, "The effects of intra-articular glucocorticoids and exercise on pain and synovitis assessed on static and dynamic magnetic resonance imaging in knee osteoarthritis: exploratory outcomes from a randomized controlled trial", *Osteoarthritis and Cartilage,* vol. 25, no. 4, pp. 481-491. |
| Rolfson, O., Karrholm, J., Dahlberg, L.E. & Garellick, G. 2011, "Patient-reported outcomes in the Swedish Hip Arthroplasty Register: results of a nationwide prospective observational study", *The Journal of bone and joint surgery.British volume,* vol. 93, no. 7, pp. 867-875. |
| Rolfson, O., Bohm, E., Franklin, P., Lyman, S., Denissen, G., Dawson, J., Dunn, J., Eresian Chenok, K., Dunbar, M., Overgaard, S., Garellick, G. & LÃ¼bbeke, A. 2016, "Patient-reported outcome measures in arthroplasty registries", *Acta Orthopaedica,* vol. 87, pp. 9-23. |
| Rosenlund, S., Broeng, L., Holsgaard-Larsen, A., Jensen, C. & Overgaard, S. 2017, "Patient-reported outcome after total hip arthroplasty: comparison between lateral and posterior approach: A randomized controlled trial in 80 patients with 12-month follow-up", *Acta Orthopaedica,* vol. 88, no. 3, pp. 239-247. |
| Rosenlund, S., Broeng, L., Jensen, C., Holsgaard-Larsen, A. & Overgaard, S. 2014, "The effect of posterior and lateral approach on patient-reported outcome measures and physical function in patients with osteoarthritis, undergoing total hip replacement: A randomised controlled trial protocol", *BMC Musculoskeletal Disorders,* vol. 15, no. 1. |
| Rosenlund, S., Holsgaard-Larsen, A., Overgaard, S. & Jensen, C. 2016, "The Gait Deviation Index is associated with hip muscle strength and patient-reported outcome in patients with severe hip osteoarthritis - A cross-sectional study", *PLoS ONE,* vol. 11, no. 4. |
| Roubion, R.C., Fox, R.S., Townsend, L.A., Pollock, G.R., Leonardi, C. & Dasa, V. 2016, "Does Marital Status Impact Outcomes After Total Knee Arthroplasty?", *Journal of Arthroplasty,* vol. 31, no. 11, pp. 2504-2507. |
| Russell, L.B., Sawatzky, R., Goldsmith, L., Lix, L.M., Sajobi, T.T., Gadermann, A.M. & Bryan, S. 2017, "Comparing patient-reported outcomes of satisfied and notsatisfied total knee arthroplasty patients: An analysis of differential item functioning", *Quality of Life Research,* vol. 26, no. 1, pp. 35-36. |
| Sadosky, A.B., Bushmakin, A.G., Cappelleri, J.C. & Lionberger, D.R. 2010, "Relationship between patient-reported disease severity in osteoarthritis and self-reported pain, function and work productivity", *Arthritis Research and Therapy,* vol. 12, no. 4. |
| Savannah, S., Bido, J., Jamie, C., Heidi, Y., Jeffrey, K. & Elena, L. 2017, "Impact of preoperative opioid use on total knee arthroplasty outcomes", *Journal of Bone and Joint Surgery - American Volume,* vol. 99, no. 10, pp. 803-808. |
| Schache, M.B., McClelland, J.A. & Webster, K.E. 2016, "Does the addition of hip strengthening exercises improve outcomes following total knee arthroplasty? A study protocol for a randomized trial", *BMC Musculoskeletal Disorders,* vol. 17, no. 1. |
| Schotanus, M.G.M., Bemelmans, Y.F.L., van der Kuy, P.H.M., Jansen, J. & Kort, N.P. 2017, "No advantage of adrenaline in the local infiltration analgesia mixture during total knee arthroplasty", *Knee Surgery, Sports Traumatology, Arthroscopy,* vol. 25, no. 9, pp. 2778-2783. |
| Schotanus, M.G.M., Pilot, P., Vos, R. & Kort, N.P. 2017, "No difference in joint awareness after mobile- and fixed-bearing total knee arthroplasty: 3-year follow-up of a randomized controlled trial", *European Journal of Orthopaedic Surgery and Traumatology,* vol. 27, no. 8, pp. 1151-1155. |
| Scott, C.E., Murray, R.C., MacDonald, D.J. & Biant, L.C. 2014, "Staged bilateral total knee replacement: changes in expectations and outcomes between the first and second operations", *The bone & joint journal,* vol. 96-B, no. 6, pp. 752-758. |
| Scott, C.E.H., Turnbull, G.S., MacDonald, D. & Breusch, S.J. 2017, "Activity levels and return to work following total knee arthroplasty in patients under 65 years of age", *The bone & joint journal,* vol. 99-B, no. 8, pp. 1037-1046. |
| Sillesen, N.H., Greene, M.E., Nebergall, A.K., Huddleston, J.I., Emerson, R., Gebuhr, P., Troelsen, A. & Malchau, H. 2016, "3-year follow-up of a long-term registry-based multicentre study on vitamin E diffused polyethylene in total hip replacement", *Hip international : the journal of clinical and experimental research on hip pathology and therapy,* vol. 26, no. 1, pp. 97-103. |
| Skinner, D., Tadros, B.J., Bray, E., Elsherbiny, M. & Stafford, G. 2016, "Clinical outcome following primary total hip or knee replacement in nonagenarians", *Annals of the Royal College of Surgeons of England,* vol. 98, no. 4, pp. 258-264. |
| Skou, S.T., Rasmussen, S., Laursen, M.B., Rathleff, M.S., Arendt-Nielsen, L., Simonsen, O. & Roos, E.M. 2016, "The two-year efficacy of 12-weeks non-surgical treatment for patients not eligible for total knee replacement-a pre-defined analysis from a randomized controlled trial", *Osteoarthritis and Cartilage,* vol. 24, pp. S183. |
| Smith, S.L., Woodburn, J. & Steultjens, M.P. 2016, "Electromechanical delay and rate of force development in individuals with knee osteoarthritis", *Osteoarthritis and Cartilage,* vol. 24, pp. S122. |
| Smith, S.R., Yang, H.Y., Collins, J.E., Katz, J.N. & Losina, E. 2016, "Impact of preoperative opioid use on total knee arthroplasty outcomes", *Arthritis and Rheumatology,* vol. 68, pp. 4004-4005. |
| Smith, S.R., Collins, J.E., Yang, H., Katz, J.N., Losina, E. & Bido, J. 2017, "Impact of Preoperative Opioid Use on Total Knee Arthroplasty Outcomes", *Journal of Bone & Joint Surgery, American Volume,* vol. 99, no. 10, pp. 803-808. |
| Soni, A., Joshi, A., Mudge, N., Wyatt, M. & Williamson, L. 2012, "Supervised exercise plus acupuncture for moderate to severe knee osteoarthritis: A small randomised controlled trial", *Acupuncture in Medicine,* vol. 30, no. 3, pp. 176-181. |
| Stambough, J.B., Xiong, A., Baca, G.R., Wu, N., Callaghan, J.J. & Clohisy, J.C. 2016, "Preoperative Joint Space Width Predicts Patient-Reported Outcomes After Total Hip Arthroplasty in Young Patients", *Journal of Arthroplasty,* vol. 31, no. 2, pp. 429-433. |
| Tanaka, M., Azus, A., Pedoia, V., Gong, J. & Li, X. 2017, "R2r1r as a potential biomarker for tracking cartilage matrix changes in osteoarthritic knees after hyaluronic acid injection", *Osteoarthritis and Cartilage,* vol. 25, pp. S326-S327. |
| Tawy, G.F., Simons, M., Rowe, P.J. & Biant, L.C. 2017, "The correlation between patient reported outcome measures and advanced biomechanics in knee osteoarthritis", *Knee,* vol. 24, no. 6, pp. VI-VII. |
| Thienpont, E. & Zorman, D. 2016, "Higher forgotten joint score for fixed-bearing than for mobile-bearing total knee arthroplasty", *Knee surgery, sports traumatology, arthroscopy : official journal of the ESSKA,* vol. 24, no. 8, pp. 2641-2645. |
| Thorstensson, C., Olsson, T., Jönsson-Lundgren, M., Garellick, G. & Dahlberg, L. 2011, "Better management of osteoarthritis (BOA)", *Physiotherapy (United Kingdom),* vol. 97, pp. eS1612-eS1613. |
| Thorstensson, C.A. 2013, "Implementation of OA care path ways in Sweden: Better management of osteoarthritis (BOA)", *Annals of the Rheumatic Diseases,* vol. 72. |
| Thorstensson, C.A., Garellick, G. & Dahlberg, L.E. 2010, "Better management of osteoarthritis (BOA)", *Osteoarthritis and Cartilage,* vol. 18, pp. S140. |
| Tilbury, C., Holtslag, M.J., Tordoir, R.L., Leichtenberg, C.S., Verdegaal, S.H.M., Kroon, H.M., Fiocco, M., Nelissen, R.G.H.H. & Vliet Vlieland, T.P.M. 2016, "Outcome of total hip arthroplasty, but not of total knee arthroplasty, is related to the preoperative radiographic severity of osteoarthritis", *Acta Orthopaedica,* vol. 87, no. 1, pp. 67-71. |
| Tjørnild, M., Søballe, K., Hansen, P.M., Holm, C. & Stilling, M. 2015, "Mobile- vs. fixed-bearing total knee replacement", *Acta orthopaedica,* vol. 86, no. 2, pp. 208-214. |
| Uhrbrand, P., Ulrich, M. & Søballe, K. 2014, "Quality of life and hip function during the first month after total hip arthroplasty", *Current Orthopaedic Practice,* vol. 25, no. 3, pp. 233-237. |
| Ulivi, M., Orlandini, L.C., Meroni, V., Lombardo, M.D.M. & Peretti, G.M. 2018, "Clinical Performance, Patient Reported Outcome, and Radiological Results of a Short, Tapered, Porous, Proximally Coated Cementless Femoral Stem: Results up to Seven Years of Follow-Up", *Journal of Arthroplasty,* vol. 33, no. 4, pp. 1133-1138. |
| Van Beers, L.W.A.H., Van Oldenrijk, J., Scholtes, V.A.B., Geerdink, C.H., Niers, B.B.A.M., Runne, W., Bhandari, M. & Poolman, R.W. 2016, "Curved versus Straight Stem Uncemented Total Hip Arthroplasty Osteoarthritis Multicenter trial (CUSTOM): Design of a prospective blinded randomised controlled multicentre trial", *BMJ Open,* vol. 6, no. 3. |
| van der List, J.P., Chawla, H., Villa, J.C. & Pearle, A.D. 2016, "Different optimal alignment but equivalent functional outcomes in medial and lateral unicompartmental knee arthroplasty", *Knee,* vol. 23, no. 6, pp. 987-995. |
| Van Der Voort, P., Valstar, E.R., Kaptein, B.L., Fiocco, M., Van Der Heide, H.J.L. & Nelissen, R.G.H.H. 2016, "Comparison of femoral component migration between Refobacin bone cement R and Palacos R + G in cemented total hip arthroplasty: A randomised controlled roentgen stereophotogrammetric analysis and clinical study", *Bone and Joint Journal,* vol. 98-B, no. 10, pp. 1333-1341. |
| Van Der Weegen, W., Hoekstra, H.J., Sijbesma, T., Austen, S. & Poolman, R.W. 2012, "Hip resurfacing in a district general hospital: 6-year clinical results using the ReCap hip resurfacing system", *BMC Musculoskeletal Disorders,* vol. 13. |
| van Hove, R.P., Brohet, R.M., van Royen, B.J. & Nolte, P.A. 2016, "High correlation of the Oxford Knee Score with postoperative pain, but not with performance-based functioning", *Knee Surgery, Sports Traumatology, Arthroscopy,* vol. 24, no. 10, pp. 3369-3375. |
| Varnum, C. 2017, "Outcomes of different bearings in total hip arthroplasty - implant survival, revision causes, and patient-reported outcome", *Danish medical journal,* vol. 64, no. 3. |
| Vines, J.B., Aliprantis, A.O., Gomoll, A.H. & Farr, J. 2016, "Cryopreserved Amniotic Suspension for the Treatment of Knee Osteoarthritis", *Journal of Knee Surgery,* vol. 29, no. 6, pp. 443-450. |
| Voort, P., Valstar, E.R., Kaptein, B.L., Fiocco, M., Heide, H.J. & Nelissen, R.G. 2016, "Comparison of femoral component migration between Refobacin bone cement R and Palacos R + G in cemented total hip arthroplasty: a randomised controlled roentgen stereophotogrammetric analysis and clinical study", *Bone and joint journal,* vol. 98-B, no. 10, pp. 1333-1341. |
| Warth, L.C., Ishmael, M.K., Deckard, E.R., Ziemba-Davis, M. & Meneghini, R.M. 2017, "Do Medial Pivot Kinematics Correlate With Patient-Reported Outcomes After Total Knee Arthroplasty?", *Journal of Arthroplasty,* vol. 32, no. 8, pp. 2411-2416. |
| Wautier, D. & Thienpont, E. 2017, "Changes in anteroposterior stability and proprioception after different types of knee arthroplasty", *Knee surgery, sports traumatology, arthroscopy : official journal of the ESSKA,* vol. 25, no. 6, pp. 1792-1800. |
| White, D.K., Lee, J., Song, J., Chang, R.W. & Dunlop, D. 2017, "Potential Functional Benefit From Light Intensity Physical Activity in Knee Osteoarthritis", *American Journal of Preventive Medicine,* vol. 53, no. 5, pp. 689-696. |
| White, S.H., Roberts, S. & Kuiper, J.H. 2015, "The cemented twin-peg Oxford partial knee replacement survivorship: a cohort study", *The Knee,* vol. 22, no. 4, pp. 333-337. |
| Whitehouse, M.R., Aquilina, A.L., Patel, S., Eastaugh-Waring, S.J. & Blom, A.W. 2013, "Survivorship, patient reported outcome and satisfaction following resurfacing and total hip arthroplasty", *Journal of Arthroplasty,* vol. 28, no. 5, pp. 842-848. |
| Whitehouse, M.R., Stefanovich-Lawbuary, N.S., Brunton, L.R. & Blom, A.W. 2013, "The impact of leg length discrepancy on patient satisfaction and functional outcome following total hip arthroplasty", *The Journal of arthroplasty,* vol. 28, no. 8, pp. 1408-1414. |
| Williams, D.P., Price, A.J., Beard, D.J., Hadfield, S.G., Arden, N.K., Murray, D.W. & Field, R.E. 2013, "The effects of age on patient-reported outcome measures in total knee replacements", *The bone & joint journal,* vol. 95-B, no. 1, pp. 38-44. |
| Wood, L.R.J., Blagojevic-Bucknall, M., Stynes, S., D'Cruz, D., Mullis, R., Whittle, R., Peat, G. & Foster, N.E. 2016, "Impairment-targeted exercises for older adults with knee pain: A proof-of-principle study (TargET-Knee-Pain)", *BMC Musculoskeletal Disorders,* vol. 17, no. 1. |
| Yu, S., Wisniewski, H.-., Lee, E., Deakin, T., Zhou, X.S., Karia, R., Strauss, E., Jazrawi, L., Iorio, R. & Band, P. 2016, "A pragmatic knee preservation registry to follow synovial fluid biomarkers and clinical outcome in patients with degenerative joint disease", *Journal of Orthopaedic Research,* vol. 34. |
| Yun, S.T., Kim, B.K., Ahn, B.M. & Oh, K.J. 2018, "Difference in the degree of improvement in patient-reported outcomes after total knee arthroplasty between octogenarians and sexagenarians: a propensity score matching analysis", *Aging Clinical and Experimental Research,* , pp. 1-6. |
| Zengerink, I., Duivenvoorden, T., Niesten, D., Verburg, H., Bloem, R. & Mathijssen, N. 2015, "Obesity does not influence the outcome after unicompartmental knee arthroplasty", *Acta Orthopaedica Belgica,* vol. 81, no. 4, pp. 776-783. |
| Zuiderbaan, H.A., van der List, J.P., Khamaisy, S., Nawabi, D.H., Thein, R., Ishmael, C., Paul, S. & Pearle, A.D. 2017, "Unicompartmental knee arthroplasty versus total knee arthroplasty: Which type of artificial joint do patients forget?", *Knee Surgery, Sports Traumatology, Arthroscopy,* vol. 25, no. 3, pp. 681-686. |
| Zywiel, M.G., Okrainec, A., Penner, T., Kassam, H., Perruccio, A., Jackson, T., Urbach, D. & Gandhi, R. 2014, "The impact of gastric bypass surgery compared to total knee arthroplasty for osteoarthritis on knee symptoms", *Osteoarthritis and Cartilage,* vol. 22, pp. S405. |
| **Prediction study** |
| Aalund, P.K., Glassou, E.N. & Hansen, T.B. 2017, "The impact of age and preoperative health-related quality of life on patient-reported improvements after total hip arthroplasty", *Clinical Interventions in Aging,* vol. 12, pp. 1951-1956. |
| Arden, N.K., Kiran, A., Judge, A., Biant, L.C., Javaid, M.K., Murray, D.W., Carr, A.J., Cooper, C. & Field, R.E. 2011, "What is a good patient reported outcome after total hip replacement?", *Osteoarthritis and Cartilage,* vol. 19, no. 2, pp. 155-162. |
| Ayers, D.C., Li, W., Oatis, C., Rosal, M.C. & Franklin, P.D. 2013, "Patient-reported outcomes after total knee replacement vary on the basis of preoperative coexisting disease in the lumbar spine and other nonoperatively treated joints,the need for a musculoskeletal comorbidity index", *Journal of Bone and Joint Surgery - Series A,* vol. 95, no. 20, pp. 1833-1837. |
| Baker, P.N., Rushton, S., Jameson, S.S., Reed, M., Gregg, P. & Deehan, D.J. 2013, "Patient satisfaction with total knee replacement cannot be predicted from pre-operative variables alone: A cohort study from the National Joint Registry for England and Wales", *Bone & Joint Journal,* vol. 95-B, no. 10, pp. 1359-1365. |
| Baker, P., Petheram, T., Jameson, S., Reed, M., Gregg, P. & Deehan, D. 2012, "The association between body mass index and the outcomes of total knee arthroplasty", *The Journal of bone and joint surgery.American volume,* vol. 94, no. 16, pp. 1501-1508. |
| Baker, P.N., Deehan, D.J., Lees, D., Jameson, S., Avery, P.J., Gregg, P.J. & Reed, M.R. 2012, "The effect of surgical factors on early patient-reported outcome measures (PROMS) following total knee replacement", *Journal of Bone and Joint Surgery - Series B,* vol. 94 B, no. 8, pp. 1058-1066. |
| Berliner, J.L., Brodke, D.J., Chan, V., SooHoo, N.F. & Bozic, K.J. 2017, "Can Preoperative Patient-reported Outcome Measures Be Used to Predict Meaningful Improvement in Function After TKA?", *Clinical orthopaedics and related research,* vol. 475, no. 1, pp. 149-157. |
| Berliner, J.L., Brodke, D.J., Chan, V., SooHoo, N.F. & Bozic, K.J. 2016, "John Charnley Award: Preoperative Patient-reported Outcome Measures Predict Clinically Meaningful Improvement in Function After THA", *Clinical orthopaedics and related research,* vol. 474, no. 2, pp. 321-329. |
| Bido, J., Yang, Y.H., Collins, J.E., Dong, Y., Driscoll, D.A., Alcantara, L.A., Thornhill, T.S. & Katz, J.N. 2017, "Predictors of Patient-Reported Outcomes of Total Joint Arthroplasty in a Developing Country", *Journal of Arthroplasty,* vol. 32, no. 6, pp. 1756-1762. |
| Brisson, N.M., Stratford, P.W. & Maly, M.R. 2017, "Patient-reported outcomes interact with muscle capacity to predict two-year stair ascent performance in women with clinical knee osteoarthritis", *Osteoarthritis and Cartilage,* vol. 25, pp. S24-S25. |
| Carlesso, L.C., Hawker, G.A., Waugh, E.J. & Davis, A.M. 2016, "Disease-specific pain and function predict future pain impact in hip and knee osteoarthritis", *Clinical rheumatology,* vol. 35, no. 12, pp. 2999-3005. |
| Duivenvoorden, T., Vissers, M.M., Verhaar, J.A., Busschbach, J.J., Gosens, T., Bloem, R.M., Bierma-Zeinstra, S.M. & Reijman, M. 2013, "Anxiety and depressive symptoms before and after total hip and knee arthroplasty: a prospective multicentre study", *Osteoarthritis and cartilage,* vol. 21, no. 12, pp. 1834-1840. |
| Eneqvist, T., Nemes, S., Bülow, E., Mohaddes, M. & Rolfson, O. 2018, "Can patient-reported outcomes predict re-operations after total hip replacement?", *International orthopaedics,* vol. 42, no. 2, pp. 273-279. |
| Escobar, A., García Pérez, L., Herrera-Espiñeira, C., Aizpuru, F., Sarasqueta, C., Gonzalez Sáenz de Tejada, M., Quintana, J.M. & Bilbao, A. 2017, "Total knee replacement: Are there any baseline factors that have influence in patient reported outcomes?", *Journal of evaluation in clinical practice,* vol. 23, no. 6, pp. 1232-1239. |
| Escobar, A., Quintana, J.M., Bilbao, A., Azkárate, J., Güenaga, J.I., Arenaza, J.C. & Gutierrez, L.F. 2007, "Effect of patient characteristics on reported outcomes after total knee replacement", *Rheumatology,* vol. 46, no. 1, pp. 112-119. |
| Feng, S., Chen, S., Wang, L., Peterfy, C., Kraus, V.B., Kamath, R., Zhang, L., Luo, Y., Cui, L., Medema, J.K. & Levesque, M.C. 2017, "Biomarkers predictive of pain improvement in knee osteoarthritis subjects treated with the anti-IL-1α/β dual variable domain immunoglobulin ABT-981", *Arthritis and Rheumatology,* vol. 69. |
| Gates, L.S., Bowen, C.J. & Arden, N.K. 2016, "Can clinical foot and ankle assessments improve the prediction of patient reported outcomes in knee arthroplasty?", *Osteoarthritis and Cartilage,* vol. 24, pp. S208. |
| Gates, L.S., Bowen, C.J., Sanchez-Santos, M.T., Delmestri, A. & Arden, N.K. 2017, "Do foot & ankle assessments assist the explanation of 1 year knee arthroplasty outcomes?", *Osteoarthritis and Cartilage,* vol. 25, no. 6, pp. 892-898. |
| Ghomrawi, H.M.K., Mancuso, C.A., Dunning, A., Gonzalez Della Valle, A., Alexiades, M., Cornell, C., Sculco, T., Bostrom, M., Mayman, D., Marx, R.G., Westrich, G., O’Dell, M. & Mushlin, A.I. 2017, "Do Surgeon Expectations Predict Clinically Important Improvements in WOMAC Scores After THA and TKA?", *Clinical orthopaedics and related research,* vol. 475, no. 9, pp. 2150-2158. |
| Greene, M.E., Rolfson, O., Gordon, M., Garellick, G. & Nemes, S. 2015, "Standard Comorbidity Measures Do Not Predict Patient-reported Outcomes 1 Year After Total Hip Arthroplasty", *Clinical orthopaedics and related research,* vol. 473, no. 11, pp. 3370-3379. |
| Greene, M.E., Rolfson, O., Nemes, S., Gordon, M., Malchau, H. & Garellick, G. 2014, "Education attainment is associated with patient-reported outcomes: Findings from the Swedish hip arthroplasty register", *Clinical orthopaedics and related research,* vol. 472, no. 6, pp. 1868-1876. |
| Hamilton, D.F., Lane, J.V., Gaston, P., Patton, J.T., MacDonald, D., Simpson, A.H.R.W. & Howie, C.R. 2013, "What determines patient satisfaction with surgery? A prospective cohort study of 4709 patients following total joint replacement", *BMJ Open,* vol. 3, no. 4. |
| Hoorntje, A., Witjes, S., Koenraadt, K.L.M., Aarts, R., Weert, T.D. & van Geenen, R.C.I. 2018, "More Severe Preoperative Kellgren–Lawrence Grades of Knee Osteoarthritis were Partially Associated with Better Postoperative Patient-Reported Outcomes in TKA Patients", *Journal of Knee Surgery,* . |
| Jain, D., Bendich, I., Nguyen, L.-.L., Nguyen, L.L., Lewis, C.G., Huddleston, J.I., Duwelius, P.J., Feeley, B.T. & Bozic, K.J. 2017, "Do Patient Expectations Influence Patient-Reported Outcomes and Satisfaction in Total Hip Arthroplasty? A Prospective, Multicenter Study", *Journal of Arthroplasty,* vol. 32, no. 11, pp. 3322-3327. |
| Jameson, S.S., Mason, J.M., Baker, P.N., Elson, D.W., Deehan, D.J. & Reed, M.R. 2014, "The impact of body mass index on patient reported outcome measures (proms) and complications following primary hip arthroplasty", *Journal of Arthroplasty,* vol. 29, no. 10, pp. 1889-1898. |
| Jiang, Y., Sanchez-Santos, M.T., Judge, A.D., Murray, D.W. & Arden, N.K. 2017, "Predictors of Patient-Reported Pain and Functional Outcomes Over 10 Years After Primary Total Knee Arthroplasty: A Prospective Cohort Study", *Journal of Arthroplasty,* vol. 32, no. 1, pp. 92-100.e2. |
| Judge, A., Arden, N.K., Batra, R.N., Thomas, G., Beard, D., Javaid, M.K., Cooper, C. & Murray, D. 2013, "The association of patient characteristics and surgical variables on symptoms of pain and function over 5 years following primary hip-replacement surgery: A prospective cohort study", *BMJ Open,* vol. 3, no. 3. |
| Judge, A., Arden, N.K., Cooper, C., Kassim javaid, M., Carr, A.J., Field, R.E. & Dieppe, P.A. 2012, "Predictors of outcomes of total knee replacement surgery", *Rheumatology (United Kingdom),* vol. 51, no. 10, pp. 1804-1813. |
| Judge, A., Arden, N.K., Price, A., Glyn-Jones, S., Beard, D., Carr, A.J., Dawson, J., Fitzpatrick, R. & Field, R.E. 2011, "Assessing patients for joint replacement: Can pre-operative Oxford hip and knee scores be used to predict patient satisfaction following joint replacement surgery and to guide patient selection?", *Journal of Bone and Joint Surgery - Series B,* vol. 93 B, no. 12, pp. 1660-1664. |
| Judge, A., Batra, R.N., Thomas, G.E., Beard, D., Javaid, M.K., Murray, D.W., Dieppe, P.A., Dreinhoefer, K.E., Peter-Guenther, K., Field, R., Cooper, C. & Arden, N.K. 2014, "Body mass index is not a clinically meaningful predictor of patient reported outcomes of primary hip replacement surgery: Prospective cohort study", *Osteoarthritis and Cartilage,* vol. 22, no. 3, pp. 431-439. |
| Judge, A., Cooper, C., Arden, N.K., Williams, S., Hobbs, N., Dixon, D., Günther, K.-., Dreinhoefer, K. & Dieppe, P.A. 2011, "Pre-operative expectation predicts 12-month post-operative outcome among patients undergoing primary total hip replacement in European orthopaedic centres", *Osteoarthritis and Cartilage,* vol. 19, no. 6, pp. 659-667. |
| Kahn, T.L., Soheili, A. & Schwarzkopf, R. 2013, "Outcomes of Total Knee Arthroplasty in Relation to Preoperative Patient-Reported and Radiographic Measures: Data From the Osteoarthritis Initiative", *Geriatric Orthopaedic Surgery & Rehabilitation,* vol. 4, no. 4, pp. 117-126. |
| Kahn, T.L., Soheili, A.C. & Schwarzkopf, R. 2014, "Poor WOMAC Scores in Contralateral Knee Negatively Impact TKA Outcomes: Data From the Osteoarthritis Initiative", *Journal of Arthroplasty,* vol. 29, no. 8, pp. 1580-1585. |
| Kakar, A., Shah, D., Gogia, A., Manohar, V.R., Desai, R., Dhawan, M. & Saxena, K.K. 2017, "Does fat distribution in body correlates with radiological features of osteoarthritis?", *Indian Journal of Rheumatology,* vol. 12, no. 5, pp. S92-S93. |
| Karia, R.J., Zhou, X., Slover, J.D. & Band, P.A. 2015, "Patient specific variables influence patient reported outcome scores in TKA population", *Osteoarthritis and Cartilage,* vol. 23, pp. A350. |
| Ko, V., Naylor, J.M., Harris, I.A., Crosbie, J. & Yeo, A.E.T. 2013, "The six-minute walk test is an excellent predictor of functional ambulation after total knee arthroplasty", *BMC Musculoskeletal Disorders,* vol. 14. |
| Kobsar, D., Osis, S.T., Boyd, J.E., Hettinga, B.A. & Ferber, R. 2017, "Wearable sensors to predict improvement following an exercise intervention in patients with knee osteoarthritis", *Journal of NeuroEngineering and Rehabilitation,* vol. 14, no. 1. |
| Kobsar, D., Osis, S.T., Boyd, J.E., Hettinga, B.A. & Ferber, R. 2017, "Wearable sensors to predict response to a hip strengthening exercise intervention in patients with knee osteoarthritis", *Osteoarthritis and Cartilage,* vol. 25, pp. S23-S24. |
| Kobsar, D., Osis, S.T., Hettinga, B.A. & Ferber, R. 2015, "Gait biomechanics and patient-reported function as predictors of response to a hip strengthening exercise intervention in patients with knee osteoarthritis", *PLoS ONE,* vol. 10, no. 10. |
| Ledford, C.K., Millikan, P.D., Nickel, B.T., Green, C.L., Attarian, D.E., Wellman, S.S., Bolognesi, M.P. & Queen, R.M. 2016, "Percent Body Fat Is More Predictive of Function After Total Joint Arthroplasty Than Body Mass Index", *Journal of Bone & Joint Surgery, American Volume,* vol. 98, no. 10, pp. 849-857. |
| Lizaur-Utrilla, A., Martinez-Mendez, D., Miralles-Muñoz, F.A., Marco-Gomez, L. & Lopez-Prats, F.A. 2016, "Negative impact of waiting time for primary total knee arthroplasty on satisfaction and patient-reported outcome", *International orthopaedics,* vol. 40, no. 11, pp. 2303-2307. |
| Logerstedt, D., Zeni, J. & Snyder-Mackler, L. 2012, "Disease severity and sex differences in knee performance and self-reports in patients with knee osteoarthritis", *Osteoarthritis and Cartilage,* vol. 20, pp. S160. |
| Logerstedt, D.S., Zeni, J., Jr. & Snyder-Mackler, L. 2014, "Sex differences in patients with different stages of knee osteoarthritis", *Archives of Physical Medicine and Rehabilitation,* vol. 95, no. 12, pp. 2376-2381. |
| Loth, F.L., Giesinger, J.M., Giesinger, K., MacDonald, D.J., Simpson, A.H.R.W., Howie, C.R. & Hamilton, D.F. 2017, "Impact of Comorbidities on Outcome After Total Hip Arthroplasty", *Journal of Arthroplasty,* vol. 32, no. 9, pp. 2755-2761. |
| Mackie, A., Muthumayandi, K., Shirley, M., Deehan, D. & Gerrand, C. 2015, "Association between body mass index change and outcome in the first year after total knee arthroplasty", *Journal of Arthroplasty,* vol. 30, no. 2, pp. 206-209. |
| Mahamed, A., Kulandaivelan & Tahseen S, S. 2014, "Effect of age, sex, BMI on functional status in primary knee osteoarthritis individuals", *Indian Journal of Physiotherapy and Occupational Therapy,* vol. 8, no. 2, pp. 84-88. |
| Mannion, A.F., Impellizzeri, F.M., Naal, F.D. & Leunig, M. 2015, "Women Demonstrate More Pain and Worse Function Before THA but Comparable Results 12 Months After Surgery", *Clinical orthopaedics and related research,* vol. 473, no. 12, pp. 3849-3857. |
| Mills, K., Osis, S.T., Martin, M., Hettinga, B.A. & Ferber, R. 2014, "A preliminary study of potential indicators of standardised exercise program success in individuals with knee osteoarthritis: Is self-report enough?", *Journal of Science and Medicine in Sport,* vol. 18, pp. e89-e90. |
| Neuburger, J., Hutchings, A., Allwood, D., Black, N. & Van Der Meulen, J.H. 2012, "Sociodemographic differences in the severity and duration of disease amongst patients undergoing hip or knee replacement surgery", *Journal of Public Health (United Kingdom),* vol. 34, no. 3, pp. 421-429. |
| Oak, S.R., Strnad, G.J., O'Rourke, C., Higuera, C.A., Spindler, K.P. & Brooks, P.J. 2017, "Mid-Term Results and Predictors of Patient-Reported Outcomes of Birmingham Hip Resurfacing", *Journal of Arthroplasty,* vol. 32, no. 1, pp. 110-118. |
| Poitras, S., Wood, K.S., Savard, J., Dervin, G.F. & Beaule, P.E. 2015, "Predicting early clinical function after hip or knee arthroplasty", *Bone and Joint Research,* vol. 4, no. 9, pp. 145-151. |
| Quintana, J.M., Aguirre, U., Barrio, I., Orive, M., Garcia, S. & Escobar, A. 2012, "Outcomes after total hip replacement based on patients' baseline status: What results can be expected?", *Arthritis Care and Research,* vol. 64, no. 4, pp. 563-572. |
| Rogers, B.A., Alolabi, B., Carrothers, A.D., Kreder, H.J. & Jenkinson, R.J. 2015, "Can the pre-operative Western Ontario and McMaster score predict patient satisfaction following total hip arthroplasty?", *The bone & joint journal,* vol. 97-B, no. 2, pp. 150-153. |
| Scott, C.E.H., Oliver, W.M., MacDonald, D., Wade, F.A., Moran, M. & Breusch, S.J. 2016, "Predicting dissatisfaction following total knee arthroplasty in patients under 55 years of age", *Bone and Joint Journal,* vol. 98-B, no. 12, pp. 1625-1634. |
| Sveikata, T., Porvaneckas, N., Kanopa, P., Molyte, A., Klimas, D., Uvarovas, V. & Venalis, A. 2017, "Age, Sex, Body Mass Index, Education, and Social Support Influence Functional Results After Total Knee Arthroplasty", *Geriatric Orthopaedic Surgery and Rehabilitation,* vol. 8, no. 2, pp. 71-77. |
| Van Onsem, S., Van Der Straeten, C., Arnout, N., Deprez, P., Van Damme, G. & Victor, J. 2016, "A New Prediction Model for Patient Satisfaction After Total Knee Arthroplasty", *The Journal of arthroplasty,* vol. 31, no. 12, pp. 2660-2667.e1. |
| Wylde, V., Dixon, S. & Blom, A.W. 2012, "The Role of Preoperative Self-Efficacy in Predicting Outcome after Total Knee Replacement", *Musculoskeletal Care,* vol. 10, no. 2, pp. 110-118. |
| **Active part of treatment study** |
| Gakhal, N., Propp, R., Keshavjee, L., Hayden, L., DaSilva, S. & Ivers, N. 2016, "Audit and feedback of patient reported outcomes in knee osteoarthritis to improve management in primary care: A pilot project", *Journal of Rheumatology,* vol. 43, no. 6, pp. 1168. |
| Slover, J.D., Karia, R.J., Hauer, C., Gelber, Z., Band, P.A. & Graham, J. 2015, "Feasibility of integrating standardized patient-reported outcomes in orthopedic care", *The American Journal of Managed Care,* vol. 21, no. 8, pp. e494-e500. |
| **Other** |
| Bellamy, N., Hochberg, M., Tubach, F., Martin-Mola, E., Awada, H., Bombardier, C., Hajjaj-Hassouni, N., Logeart, I., Matucci-Cerinic, M., Van De Laar, M., Van Der Heijde, D. & Dougados, M. 2015, "Development of multinational definitions of minimal clinically important improvement and patient acceptable symptomatic state in osteoarthritis", *Arthritis Care and Research,* vol. 67, no. 7, pp. 972-980. |
| Biggs, P.R., Jones, P., Wilson, C., Watling, D., Whatling, G.M. & Holt, C.A. 2016, "Is restoration of healthy gait biomechanics following total knee replacement surgery reflected in patient reported outcome measures?", *Osteoarthritis and Cartilage,* vol. 24, pp. S117. |
| Carotti, M., Salaffi, F., Di Carlo, M. & Giovagnoni, A. 2017, "Relationship between magnetic resonance imaging findings, radiological grading, psychological distress and pain in patients with symptomatic knee osteoarthritis", *Radiologia Medica,* vol. 122, no. 12, pp. 934-943. |
| Davis, A.M., Badley, E.M., Hogg-Johnson, S., Ibrahim, S., Perruccio, A.V., Wong, R., Beaton, D.E., Côté, P., Gignac, M.A. & Streiner, D. 2010, "Recovery following total hip and knee replacement: The interplay of physical impairments, activity limitations and participation restrictions", *Osteoarthritis and Cartilage,* vol. 18, pp. S41-S42. |
| Gandhi, R., Zywiel, M.G., Mahomed, N.N. & Perruccio, A.V. 2015, "Depression and the overall burden of painful joints: An examination among individuals undergoing hip and knee replacement for osteoarthritis", *Arthritis,* vol. 2015. |
| Jameson, K., Balshaw, R., Phillips, C., Martin, G.R., Everett, S.V., Watson, D.J. & Taylor, S.D. 2011, "Inadequate pain relief in knee osteoarthritis and patient reported outcomes: A survey of osteoarthritis real world therapies (SORT) in the United Kingdom", *Value in Health,* vol. 14, no. 7, pp. A313. |
| Keurentjes, J.C. 2015, "CORR Insights^®^: Standard Comorbidity Measures Do Not Predict Patient-reported Outcomes 1 Year After Total Hip Arthroplasty", *Clinical orthopaedics and related research,* vol. 473, no. 11, pp. 3380-3382. |
| Kjærgaard, N., Kjærsgaard, J.B., Petersen, C.L., Jensen, M.U. & Laursen, M.B. 2017, "Thresholds for the Oxford Hip Score after total hip replacement surgery: a novel approach to postoperative evaluation", *Journal of Orthopaedics and Traumatology,* vol. 18, no. 4, pp. 401-406. |
| Knowles, S.R., Nelson, E.A., Castle, D.J., Salzberg, M.R., Choong, P.F.M. & Dowsey, M.M. 2016, "Using the common sense model of illness to examine interrelationships between symptom severity and health outcomes in end-stage osteoarthritis patients", *Rheumatology (United Kingdom),* vol. 55, no. 6, pp. 1066-1073. |
| Kwoh, C.K., Guehring, H., Ashbeck, E., Hannon, M.J. & Aydemir, A. 2017, "Two-year changes in knee osteoarthritis symptoms: Comparing clinical relevance of patient-reported outcomes by anchoring to knee replacement", *Arthritis and Rheumatology,* vol. 69. |
| Lee, A., Price, L.L., Driban, J., Harvey, W.F., McAlindon, T.E., Rodday, A.M. & Wang, C. 2016, "Minimally important differences for four patient-reported outcomes measurement information system (PROMIS) short forms: Physical function, pain interference, depression, and anxiety among adults with knee osteoarthritis", *Arthritis and Rheumatology,* vol. 68, pp. 376-378. |
| Lee, A.C., Driban, J.B., Price, L.L., Harvey, W.F., Rodday, A.M. & Wang, C. 2017, "Responsiveness and Minimally Important Differences for 4 Patient-Reported Outcomes Measurement Information System Short Forms: Physical Function, Pain Interference, Depression, and Anxiety in Knee Osteoarthritis", *Journal of Pain,* vol. 18, no. 9, pp. 1096-1110. |
| Lenguerrand, E., Wylde, V., Brunton, L., Gooberman-Hill, R., Blom, A. & Dieppe, P. 2016, "Selecting, assessing and interpreting measures of function for patients with severe hip pathology: The need for caution", *Orthopaedics and Traumatology: Surgery and Research,* vol. 102, no. 6, pp. 741-746. |
| Luyten, F.P., Bierma-Zeinstra, S., Dell'Accio, F., Kraus, V.B., Nakata, K., Sekiya, I., Arden, N.K. & Lohmander, L.S. 2018, "Toward classification criteria for early osteoarthritis of the knee", *Seminars in arthritis and rheumatism,* vol. 47, no. 4, pp. 457-463. |
| Mahler, E.A.M., Boers, N., Bijlsma, J.W.J., Van Den Hoogen, F.H.J., Den Broeder, A.A. & Van Den Ende, C.H.M. 2018, "Patient acceptable symptom state in knee osteoarthritis patients succeeds across different patient-reported outcome measures Assessing Physical Function, But Fails Across Other Dimensions and Rheumatic Diseases", *Journal of Rheumatology,* vol. 45, no. 1, pp. 122-127. |
| Mills, K.A.G., Naylor, J.M., Eyles, J.P., Roos, E.M. & Hunter, D.J. 2016, "Examining the minimal important difference of patient-reported outcome measures for individuals with knee osteoarthritis: A model using the knee injury and osteoarthritis outcome score", *Journal of Rheumatology,* vol. 43, no. 2, pp. 395-404. |
| Naylor, J.M., Hayen, A., Davidson, E., Hackett, D., Harris, I.A., Kamalasena, G. & Mittal, R. 2014, "Minimal detectable change for mobility and patient-reported tools in people with osteoarthritis awaiting arthroplasty", *BMC Musculoskeletal Disorders,* vol. 15, no. 1. |
| Paulsen, A., Roos, E.M., Pedersen, A.B. & Overgaard, S. 2014, "Minimal clinically important improvement (MCII) and patient-acceptable symptom state (PASS) in total hip arthroplasty (THA) patients 1 year postoperatively", *Acta Orthopaedica,* vol. 85, no. 1, pp. 39-48. |
| Rouquette, A., Blanchin, M., Sébille, V., Guillemin, F., Côté, S.M., Falissard, B. & Hardouin, J.-. 2014, "The minimal clinically important difference determined using item response theory models: An attempt to solve the issue of the association with baseline score", *Journal of clinical epidemiology,* vol. 67, no. 4, pp. 433-440. |
| Schilling, C., Dowsey, M.M., Clarke, P.M. & Choong, P.F. 2016, "Using Patient-Reported Outcomes for Economic Evaluation: Getting the Timing Right", *Value in health : the journal of the International Society for Pharmacoeconomics and Outcomes Research,* vol. 19, no. 8, pp. 945-950. |
| Terwee, C.B., Roorda, L.D., Dekker, J., Bierma-Zeinstra, S.M., Peat, G., Jordan, K.P., Croft, P. & de Vet, H.C.W. 2010, "Mind the MIC: large variation among populations and methods", *Journal of clinical epidemiology,* vol. 63, no. 5, pp. 524-534. |
| Terwee, C.B., Roorda, L.D., Knol, D.L., De Boer, M.R. & De Vet, H.C.W. 2009, "Linking measurement error to minimal important change of patient-reported outcomes", *Journal of clinical epidemiology,* vol. 62, no. 10, pp. 1062-1067. |
| Tubach, F., Ravaud, P., Baron, G., Falissard, B., Logeart, I., Bellamy, N., Bombardier, C., Felson, D., Hochberg, M., Van Der Heijde, D. & Dougados, M. 2005, "Evaluation of clinically relevant changes in patient reported outcomes in knee and hip osteoarthritis: The minimal clinically important improvement", *Annals of the Rheumatic Diseases,* vol. 64, no. 1, pp. 29-33. |
| Van Der Wees, P.J., Wammes, J.J.G., Akkermans, R.P., Koetsenruijter, J., Westert, G.P., Van Kampen, A., Hannink, G., De Waal-Malefijt, M. & Schreurs, B.W. 2017, "Patient-reported health outcomes after total hip and knee surgery in a Dutch University Hospital Setting: Results of twenty years clinical registry", *BMC Musculoskeletal Disorders,* vol. 18, no. 1. |
| Williams, D.P., Blakey, C.M., Hadfield, S.G., Murray, D.W., Price, A.J. & Field, R.E. 2013, "Long-term trends in the Oxford knee score following total knee replacement", *The bone & joint journal,* vol. 95-B, no. 1, pp. 45-51. |
| Wright, R.W. 2009, "Knee injury outcomes measures", *Journal of the American Academy of Orthopaedic Surgeons,* vol. 17, no. 1, pp. 31-39. |
| Zampelis, V., Ornstein, E., Franzén, H. & Atroshi, I. 2014, "A simple visual analog scale for pain is as responsive as the WOMAC, the SF-36, and the EQ-5D in measuring outcomes of revision hip arthroplasty", *Acta Orthopaedica,* vol. 85, no. 2, pp. 128-132. |
