# Supplementary material for: Implementing patient-reported outcomes in clinical decision-making within knee and hip osteoarthritis: an explorative review
Source: BMC Musculoskelet Disord. 2019 May 17;20:230. doi: 10.1186/s12891-019-2620-2 (PMC6525425; doi:10.1186/s12891-019-2620-2)
Supplement: Supplementary file 3 — is a .doc file which describes the included patient-reported outcome measures, abbreviations and classifications of the PROMs as generic, disease-specific or domain-specific questionnaires. (DOCX 35 kb) [file 12891_2019_2620_MOESM3_ESM.docx]

# Additional file 3

The table describes the included patient-reported outcome measures (PROMs), abbreviations and classifications of PROMs as generic, disease-specific or domain-specific questionnaires.

| **Patient-reported outcome measure** | **Abbreviation** | **Generic** | **Disease-specific** | **Domain-specific** |
| --- | --- | --- | --- | --- |
| European Quality Of Life - 5 Dimensions | EQ-5D | X |  |  |
| Short Form 12 | SF-12 | X |  |  |
| Short Form 36 | SF-36 | X |  |  |
| Patient-Reported Outcomes Measurement Information System | PROMIS | X |  |  |
| Single Assessment Numeric Evaluation | SANE | X |  |  |
| Patient Global Impression of Severity/Change | PGI-S/C | X |  |  |
| Western Ontario and McMaster Universities Osteoarthritis Index | WOMAC |  | X |  |
| Knee Injury and Osteoarthritis Outcome Score | KOOS |  | X |  |
| Oxford Knee Score | OKS |  | X |  |
| Knee Society Score | KSS |  | X |  |
| Oxford Hip Score | OHS |  | X |  |
| Hip Disability and Osteoarthritis Outcome Score | HOOS |  | X |  |
| Visual Analogue Scale | VAS |  |  | X |
| Harris Hip Score | HHS |  | X |  |
| Forgotten Joint Score | FJS-12 |  | X |  |
| Lower Extremity Functional Scale | LEFS |  | X |  |
| Tegner Lysholm Knee Scoring Scale | TLKS |  | X |  |
| International Knee Documentation Comittee | IKDC |  | X |  |
| Brief Pain Inventory | BPI |  |  | X |
| Arthritis Self-Efficacy Scale | ASES |  | X |  |
| Knee Outcome Survey Score | KOS |  | X |  |
| Patients Global Assessment of Pain (Osteoarthritis Modular Measurement System) | PGAP (O2MS) |  | X |  |
| The Measurement of Intermittent and Constant Osteoarthritis Pain | ICOAP |  | X |  |
| Lequesne Algofunctional Indexes | LAI |  | X |  |
| High-Activity Arthroplasty Score | HAAS |  | X |  |
| Numerical Rating Scale | NRS |  |  | X |
| Japanese Orthopaedic Association Hip Disease Evaluation | JHEQ |  | X |  |
| Anterior Knee Pain Scale (Kujala) | AKPS |  | X |  |
| The Osteoarthritis Knee and Hip Quality of Life Questionnaire (Arthrose des Membred Inférieurs et Qualité De Vie) | OAHQOL (AMIQUAL) |  | X |  |
| Paindetect Questionnaire | PD-Q |  |  | X |
| Hip Outcome Survey Score | HOS |  | X |  |
| The International Hip Outcome Tool-33 | IHOT-33 |  | X |  |
| Pain Impact Questionnaire | PIQ |  |  | X |
| Physical Activity Scale For The Elderly | PASE |  |  | X |
| Chronic Pain Grade Scale | CPGS |  |  | X |
| Late-Life Function and Disability Instrument | LATE-LIFE DFI |  |  | X |
| Activity Scale for Arthroplasty Patients | ASAP |  | X |  |
| The Osteoarthritis Quality of Life Scale | OAQOL |  | X |  |
| **Total number of PROMs** | **38** | **6** | **24** | **8** |
